# Primary biliary cirrhosis and inflammatory bowel disease: a two-sample bidirectional Mendelian randomization study

**DOI:** 10.3389/ebm.2026.10940

**Published:** 2026-09-17

**Authors:** Mingyi Yang, Jiale Xie, Jing Hu, Pengfei Wen, Lin Liu, Zhi Yang, Ming Zhang, Changliang Zhu, Yani Su

**Affiliations:** 1 Department of Joint Surgery, Honghui Hospital, Xi’an Jiaotong University, Xi’an, Shaanxi, China; 2 Department of Intensive Care Unit, Honghui Hospital, Xi’an Jiaotong University, Xi’an, Shaanxi, China; 3 Department of Radiotherapy, Tangdu Hospital, The Fourth Military Medical University, Xi’an, Shaanxi, China; 4 Department of General Practice, Honghui Hospital, Xi’an Jiaotong University, Xi’an, Shaanxi, China

**Keywords:** bidirectional, causality, genetic, inflammatory bowel disease, primary biliary cirrhosis

## Abstract

Observational studies have frequently reported an association between primary biliary cirrhosis (PBC) and inflammatory bowel disease (IBD). In this study, we leveraged summary-level data from genome-wide association studies (GWAS) to conduct a two-sample bidirectional Mendelian randomization (MR) analysis, with the primary objective of investigating the genetic causal relationship between PBC and IBD, comprising ulcerative colitis (UC) and Crohn’s disease (CD). Additionally, a validation analysis was performed by repeating the bidirectional MR framework, alternately defining PBC and IBD as the exposure and outcome variables to confirm the directionality of the observed associations. A comprehensive panel of sensitivity analyses was implemented to test the robustness of the findings. We first examined the genetic causality at the subtype level. In the forward MR, PBC exerted a significant positive causal effect on UC (P < 0.001, OR 95% CI = 1.081 [1.037–1.127]) and on CD (P = 0.002, OR 95% CI = 1.136 [1.047–1.232]). In the reverse MR, only UC showed a significant negative causal influence on PBC (P > 0.003, OR 95% CI = 0.788 [0.672–0.924]), whereas no significant effect was detected from CD on PBC (P = 0.431, OR 95% CI = 0.956 [0.856–1.068]). We then extended the analysis to the overall IBD phenotype. The forward MR demonstrated a significant positive genetic relationship between PBC on IBD (P < 0.001, OR 95% CI = 1.076 [1.042–1.110]). Conversely, the reverse MR did not support a causal effect of IBD on PBC (P = 0.357, OR 95% CI = 0.898 [0.714–1.129]). The robustness of all these findings was confirmed by comprehensive sensitivity analyses, which showed no evidence of heterogeneity, horizontal pleiotropy, or undue influence from individual instrumental variables. Our MR analysis demonstrates that PBC serves as a genetic determinant of IBD as a whole and of UC/CD separately, whereas reverse causation is limited to a protective effect of UC on PBC. This direction-dependent and subtype-specific causal architecture provides novel insights into the shared etiological pathways between PBC and IBD.

## Highlights

- Forward MR consistently demonstrates positive genetic causal effects of PBC on UC, on CD, and on overall IBD, positioning PBC as a genetic risk factor across the IBD spectrum and justifying enhanced clinical monitoring for IBD manifestations in PBC patients.
- Reverse MR yields asymmetric findings: a significant protective genetic effect of UC on PBC is observed, but no such effect is found for CD on PBC, nor for overall IBD on PBC, indicating that the influence of IBD on PBC is uniquely attributable to UC.
- These results delineate a directional and subtype-specific genetic architecture in which PBC exerts broad causal effects on both IBD subtypes, while only UC exerts a counteracting effect on PBC, with no reciprocal influence from overall IBD.

## Impact statement

This study elucidated the causal association between primary biliary cirrhosis and inflammatory bowel disease at the genetic level employing a two-sample bidirectional Mendelian randomization analysis. The forward Mendelian randomization analysis yielded compelling evidence of a positive genetic causal linkage between primary biliary cirrhosis and both ulcerative colitis and Crohn’s disease (or inflammatory bowel disease). Primary biliary cirrhosis emerges as a genetic predisposing factor for the development of ulcerative colitis and Crohn’s disease (or inflammatory bowel disease). Consequently, clinical vigilance for the manifestation of ulcerative colitis and Crohn’s disease should be heightened among individuals diagnosed with primary biliary cirrhosis, suggesting a potential shift towards routine screening protocols for ulcerative colitis and Crohn’s disease (or inflammatory bowel disease) in primary biliary cirrhosis patient care. Conversely, the reverse Mendelian randomization analysis delineated a negative genetic causality between ulcerative colitis and primary biliary cirrhosis. Notably, there was no discernible genetic causality detected between Crohn’s disease (or inflammatory bowel disease) and primary biliary cirrhosis. Ulcerative colitis possibly exerting a genetic protective effect against primary biliary cirrhosis, its imply that individuals with ulcerative colitis might be less susceptible to primary biliary cirrhosis development.

## Introduction

Inflammatory bowel disease (IBD), encompassing ulcerative colitis (UC) and Crohn’s disease (CD), is characterized by chronic, progressive immune-mediated inflammation of the gastrointestinal tract [1]. While IBD can manifest at any age, its incidence peaks during early adulthood, predominantly affecting children, adolescents, and young adults. In North America, the annual incidence of UC ranges from 0 to 19.2 per 100,000 individuals, whereas in Europe it ranges from 0.6 to 24.3 per 100,000 individuals. Similarly, the incidence of CD in North America spans from 0 to 20.2 per 100,000 individuals, and in Europe, from 0.3 to 12.7 per 100,000 individuals [2]. The prevalence of UC varies from 7.6 to 246.0 cases per 100,000 individuals per year, while the prevalence of CD ranges from 3.6 to 214.0 cases per 100,000 individuals per year [3]. There is no known cure for IBD, however, symptoms can be managed through the administration of anti-inflammatory steroids or immunosuppressants to reduce inflammation, dietary modifications to eliminate potential environmental triggers, and, in severe cases, surgical intervention to excise damaged sections of the intestine [4]. IBD significantly impairs patients’ quality of life and is associated with elevated risks of hospitalization and surgery [1]. Despite extensive efforts to enhance treatment strategies and broaden therapeutic targets, IBD remains a debilitating condition with the potential to progress and lead to irreversible complications. One limitation of current treatment modalities is that they target dysregulated inflammatory pathways without fully restoring the underlying pathological processes to a pre-disease state [5]. Emerging evidence supports the existence of a preclinical stage of IBD, during which immune and inflammatory pathways are altered prior to clinical diagnosis. Advancing understanding of this pre-diagnosis phase presents opportunities for disease prediction, prevention, and interception. Targeting early causative events in IBD development can potentially prevent or mitigate disease onset, offering a substantive opportunity for disease modification [5].

IBD is believed to arise from an inappropriate and persistent inflammatory response by a genetically susceptible host to symbiotic microorganisms [6]. The mechanism underlying IBD involves an uncontrolled, immune-mediated inflammatory response to an unidentified environmental trigger that interacts with the gut microbiome, primarily affecting the digestive tract [2, 7, 8]. The pathogenesis of IBD is complex, resulting from the interaction of the immune system, gut microbiota, and genetic factors. These components may be influenced by various environmental factors that shift the balance of the gut and certain parenteral tissues towards a pro-inflammatory state. Under normal conditions, the body exhibits immune tolerance to the host microbiome; however, external influences can disrupt this balance, inducing a hyperimmune response that damages the gastrointestinal mucosa [3]. Notably, over 50% of IBD susceptibility loci are also associated with other inflammatory and autoimmune diseases [6]. For instance, the same coding variant of PTPN22 (R620W) serves as a significant risk factor for type 1 diabetes and rheumatoid arthritis but has a protective effect against CD [9]. Both UC and primary sclerosing cholangitis (PSC) share loci such as MST1, IL2, CARD9, and REL [10]. Moreover, risk loci for CD unexpectedly overlap with regions susceptible to *Mycobacterium leprae* infection, including genes such as NOD2, C13orf31, and LRRK2 [11]. This overlap may facilitate the identification of risk or protective factors for IBD. Consequently, studying the relationship between IBD and other diseases is of significant importance for the effective management of IBD.

Primary biliary cirrhosis (PBC) is an autoimmune liver disease characterized by the presence of highly specific anti-mitochondrial antibodies (AMAs) and autoreactive T cells in the serum. This disease causes progressive destruction of the intrahepatic bile ducts, leading to chronic cholestasis, portal vein inflammation, and fibrosis, which can culminate in cirrhosis and eventually liver failure. PBC predominantly affects women, with diagnoses typically occurring in their 50s and 60s [12]. It is most prevalent in Northern European countries, such as England and Scotland, and in the northern United States, particularly Minnesota [13, 14]. A systematic review suggests that the prevalence of PBC ranges from 7 to 400 cases per million individuals [15]. Although PBC manifests in various geographic locations, evidence indicates that its prevalence and incidence are increasing. The identification of geographic clusters of PBC suggests significant genetic and environmental contributions to its development [16]. The concordance rate, family prevalence, and genetic associations observed in identical twins further underscore the importance of genetic factors in PBC [17]. Previous studies have demonstrated a close relationship between PBC and IBD, suggesting mutual influences [18, 19]. The reduced bile flow resulting from PBC’s immune-mediated tissue damage and the accumulation of toxic bile products not only perpetuate biliary epithelial damage but also alter the composition of the gut and biliary microbiota and their interactions with the host. The polyclonal high IgM response and autoantibodies in PBC consistently cross-react with microbial antigens in IBD, leading to changes in the composition of the gut or biliary tract microbiota and subsequent alterations in epithelial integrity, which promote microbial translocation [18]. However, the causal relationship between IBD and PBC remains unclear. Investigating this causal relationship is of significant importance for clinical prevention and treatment strategies.

Genome-wide association study (GWAS) is a research method that detects genetic variations across the entire genome in a large population to identify the genetic loci associated with specific diseases or traits. In recent years, the implementation of large-scale GWAS has generated a vast amount of summary statistics, providing a crucial data foundation for Mendelian randomization (MR) analysis. MR presents a pioneering statistical approach employing genetic variants, commonly single nucleotide polymorphisms (SNPs), as instrumental variables (IVs) to investigate the impact of modifiable exposures on outcomes and to evaluate the causal relationship between exposure and outcome at the genetic level. The random distribution of genetic variation at conception minimizes potential biases stemming from confounding variables and reverse causation, thereby enhancing the robustness of findings in MR analysis [20]. The stochastic nature of genetic variation during gametophyte formation, impervious to environmental influences, enables MR analysis to emulate randomized controlled trials (RCTs) to a certain extent [21]. Consequently, MR analysis has gained widespread adoption in the causal assessment of diseases in recent years [22–24]. In this study, we conducted a two-sample bidirectional MR analysis utilizing aggregated summary data from an openly accessible genome-wide association study (GWAS) to elucidate the genetic causality between PBC and IBD. Our study adheres to the STROBE-MR guidelines for the reporting of MR, the STROBE-MR checklist of our study is shown in Supplementary Table S1.

## Materials and methods

### Study design

We conducted a two-sample bidirectional MR analysis to explore the genetic underpinnings of the causal relationship between PBC and IBD. Initially, a forward MR analysis was executed, wherein PBC served as the exposure variable, while UC and CD were considered as outcome variables. Subsequently, a reverse MR analysis was undertaken, with UC and CD as exposure variables and PBC as the outcome variable. The validation analysis conducted a bidirectional MR analysis, treating PBC and IBD as the exposure and outcome variables respectively. Our genetic causal assessment relied upon three fundamental assumptions of MR analysis: 1) the IVs are strongly correlated with the exposure variables, 2) the IVs are not associated with the outcome variables or confounding factors, and 3) the IVs solely influence outcomes through the exposure [23]. The GWAS summary data for both exposures and outcomes utilized in this investigation were sourced from publicly accessible databases, thus obviating the necessity for ethical statements and informed consent. Detailed information regarding the data employed in this study can be found in Supplementary Table S2.

### Data source

The GWAS summary data for PBC were retrieved from the IEU OpenGWAS database. This dataset comprised 11,375 individuals of European descent, consisting of 2,861 PBC cases and 8,514 controls, and encompassed 119,756 SNPs. Participants self-reported British or Irish ancestry. Diagnosis of PBC adhered to established criteria, including serological evidence of AMAs at a titer of 1:40 or higher, liver biopsy findings consistent with PBC pathology, and biochemical markers indicative of the condition [25]. Genotyping of participants utilized the Illumina iSelect HD custom genotyping array, followed by the extraction of normalized probe intensities adhering to predefined laboratory quality control metrics, and genotype assignments conducted using optiCall software [26]. Further detailed information regarding the dataset can be found in the associated publication [25]. Conversely, GWAS summary data for UC and CD were sourced from the Finnish consortium. The UC dataset included 214,620 European participants, with 4,320 cases and 210,300 controls, encompassing 16,380,459 SNPs. The CD dataset comprised 211,107 European participants, with 807 cases and 210,300 controls, and included 16,380,453 SNPs. The IBD GWAS dataset was derived from a European-ancestry cohort of 218,792 individuals, comprising 5,673 cases and 213,119 controls, and provided genotypic information for 16,380,466 SNPs. Case identification was facilitated through the application of the M13 code within the International Classification of Diseases-Tenth Revision (ICD-10) classification system. Genotyping utilized Illumina and Affymetrix chip arrays (Illumina Inc., San Diego, CA, USA, and Thermo Fisher Scientific, Santa Clara, CA, USA, respectively). Researchers interested in further elucidation regarding the datasets employed are encouraged to consult the FinnGen website for Supplementary Material.

### IVs selection

Ensuring the robustness and credibility of MR analysis outcomes hinges upon rigorous selection criteria for IVs. This study implemented a comprehensive IV screening protocol. Initially, SNPs exhibiting significant correlations with the exposure of interest were identified from the summary data of GWAS, employing a stringent correlation threshold of P < 5 × 10^-8^. Subsequently, a criterion of F statistic >10 was applied to ascertain strong correlation, calculated using the formula: F = R2(N-K-1)/K(1-R2). Should the prerequisite number of SNPs for MR analysis not be attainable under these conditions, adjustments to the correlation threshold were informed by pertinent literature [27–29]. In the subsequent phase, potential biases stemming from linkage disequilibrium (LD) among SNPs were addressed by eliminating those exhibiting strong LD, defined by an r2 < 0.001 and a clumping distance of 10,000 kb. Following this, SNPs meeting the criteria were cross-referenced with GWAS summary data related to the outcome variable, supplemented by proxy SNP identification via online LD link platform when necessary. Palindromic SNPs, susceptible to confounding due to intermediate allele frequencies, were then systematically removed. Furthermore, SNPs exhibiting correlations with the outcome variable were excised using thresholds consistent with those employed for the exposure variable. Finally, potential confounding factors were meticulously considered and adjusted for in both forward and reverse MR analyses. In the forward MR analyses investigating the causal relationship between PBC as exposure and UC as outcome, confounding variables among which were familial history of IBD, antecedent gastrointestinal infections, and the utilization of oral contraceptives [30]. And the forward MR analyses of CD as outcome, confounding variables includes smoking, family history of IBD, antecedent gastrointestinal infections, and the utilization of oral contraceptives [31]. In parallel, in the reverse MR analyses exploring UC or CD as the exposure and PBC as the outcome, confounding variables encompassed variables such as smoking, history of infections, and hormone replacement therapy [17, 32, 33].

### MR analysis

We employed a diverse array of MR analysis to comprehensively evaluate the genetic causality between exposure and outcome variables. Our primary analytical approach was the random-effects inverse variance weighted (IVW) method, widely recognized as the most statistically robust technique in MR analysis. Additionally, we employed four supplementary MR analysis methods: MR Egger, weighted median, simple mode, and weighted mode. Furthermore, three MR analysis methods—maximum likelihood, penalized weighted median, and fixed effects IVW—were utilized for validation results. Consistent with established MR analysis literature, random-effects IVW represents the gold standard in MR analysis. In instances where the findings of alternative MR methods diverged from those of random-effects IVW, precedence was given to the conclusions drawn from random-effects IVW analysis [20–22]. The IVW method offers the most precise estimates under the assumption of instrument validity [23]. However, it is important to note that in the presence of horizontal pleiotropy, the IVW method may produce biased estimates of causal effects [34].

### Sensitivity analysis

To ensure the robustness of our MR analysis findings, we conducted a comprehensive set of sensitivity analyses. Initially, we executed tests to evaluate heterogeneity and horizontal pleiotropy. Heterogeneity was assessed using Cochran’s Q statistic for MR-IVW and Rucker’s Q statistic for MR Egger. For horizontal pleiotropy assessment, we employed the intercept test of MR Egger and the global test of MR Pleiotropy Residual Sum and Outlier (MR-PRESSO). Subsequently, outlier detection was conducted, employing the radial variants of IVW and MR Egger, and integrate a more robust distortion test from MR-PRESSO. It is imperative to highlight that identified outliers necessitate exclusion followed by reassessment to ascertain genetic causality. Additionally, we performed Leave-one-out analyses to identify individual SNPs influencing MR analysis outcomes. Any single SNPs found to exert influence on MR results underwent exclusion and subsequent reevaluation for genetic causality. Lastly, we subjected the MR analysis to a normal distribution test using MR Robust Adjusted Profile Score (MR-RAPS).

### Statistical analysis

The statistical analyses in this study were conducted using R version 4.1.2. Genetic causal assessment was performed utilizing the “TwoSampleMR” software package within the R environment. A significance threshold of P < 0.05 was applied to determine genetic causality. Specifically, positive genetic causality was inferred when the odds ratio (OR) > 1 at P < 0.05, whereas negative genetic causality was indicated by an OR < 1 at P < 0.05. Heterogeneity and horizontal pleiotropy were assessed using significance thresholds, whereby P > 0.05 suggested the absence of these phenomena. Additionally, the normal distribution assumption was evaluated, with a P > 0.05 indicating compliance with this assumption.

## Results

### Genetic causality between PBC on UC

After accounting for LD, we identified 22 SNPs exhibiting a strong correlation with PBC. These SNPs are all present in the GWAS summary data for UC. Notably, there were no palindromic SNPs among them, and none of the 22 SNPs demonstrated an association with UC. It is also important to highlight that there was no confounding SNPs detected. Consequently, these 22 SNPs were utilized as IVs for assessing the genetic causality between PBC on UC (Supplementary Table S3).

Random-effects IVW results indicated a positive genetic causality between PBC on UC (P = 0.024, OR = 1.065, 95% confidence interval [CI] = 1.008–1.124). Additional supplementary methods, except MR Egger, yielded consistent findings with the random-effects IVW results (Supplementary Figures S1, S2A). Consistency was also observed across three verification methods, reinforcing the random-effects IVW analysis (Supplementary Figure S1). Cochran’s Q statistic for MR-IVW and Rucker’s Q statistic for MR Egger analysis demonstrated heterogeneity in the genetic causal assessment of PBC on UC (P < 0.05). While MR Egger’s intercept test did not reveal horizontal pleiotropy (P > 0.05), the global test of MR-PRESSO did (P < 0.05) (Supplementary Table S7). Examination of radial variants in the IVW and MR Egger plots identified outliers in the MR analysis (Supplementary Figure S2B). The distortion test of MR-PRESSO identified two significant outliers (rs522127, rs8067378) (Supplementary Table S7). Leave-one-out analysis indicated that genetic causal assessment was influenced by a single SNP (rs7775055) (Supplementary Figure S2C). MR-RAPS analysis supported a normal distribution (P > 0.05) (Supplementary Figure S2D; Supplementary Table S7). A second round of MR analysis was conducted after removing the two identified outliers. Random-effects IVW results confirmed a positive genetic causal relationship between PBC on UC (P = 0.004, OR = 1.069, 95% CI = 1.022–1.119). Among the four supplementary methods, MR Egger and the simple mode produced results contrary to the random-effects IVW findings, whereas the weighted median and weighted mode analyses supported the random-effects IVW results (Supplementary Figures S1, S3A). Consistency was maintained across three verification methods (Supplementary Figure S1). Heterogeneity persisted in the genetic causal assessment (P < 0.05). MR Egger’s intercept test again did not detect horizontal pleiotropy (P > 0.05), while the MR-PRESSO global test did (P < 0.05) (Supplementary Table S7). Radial variant analysis in IVW and MR Egger plots continued to reveal outliers (Supplementary Figure S3B). The MR-PRESSO distortion test detected one significant outlier (rs72678531) (Supplementary Table S7). Leave-one-out analysis showed that the genetic causal assessment was not affected by individual SNPs (Supplementary Figure S3C). MR-RAPS analysis continued to support a normal distribution (P > 0.05) (Supplementary Figure S3D; Supplementary Table S7).

Finally, a third round of MR analysis was conducted after the removal of one outlier. The random-effects IVW results indicated a positive genetic causality between PBC on UC (P < 0.001, OR 95% CI = 1.081 [1.037–1.127]). Among the supplementary methods, all except MR Egger corroborated the random-effects IVW findings (Figures 1, 2A). Additionally, the three verification methods were consistent with the random-effects IVW results (Figure 1). There was no evidence of heterogeneity or horizontal pleiotropy in the genetic causal assessment (P > 0.05) (Table 1). Although the radial variants of IVW and MR Egger identified outliers (Figure 2B), the MR-PRESSO distortion test detected no outliers (Table 1). The analysis was not influenced by single SNPs (Figure 2C), and showed a normal distribution (P > 0.05) (Figure 2D; Table 1).

**FIGURE 1:**
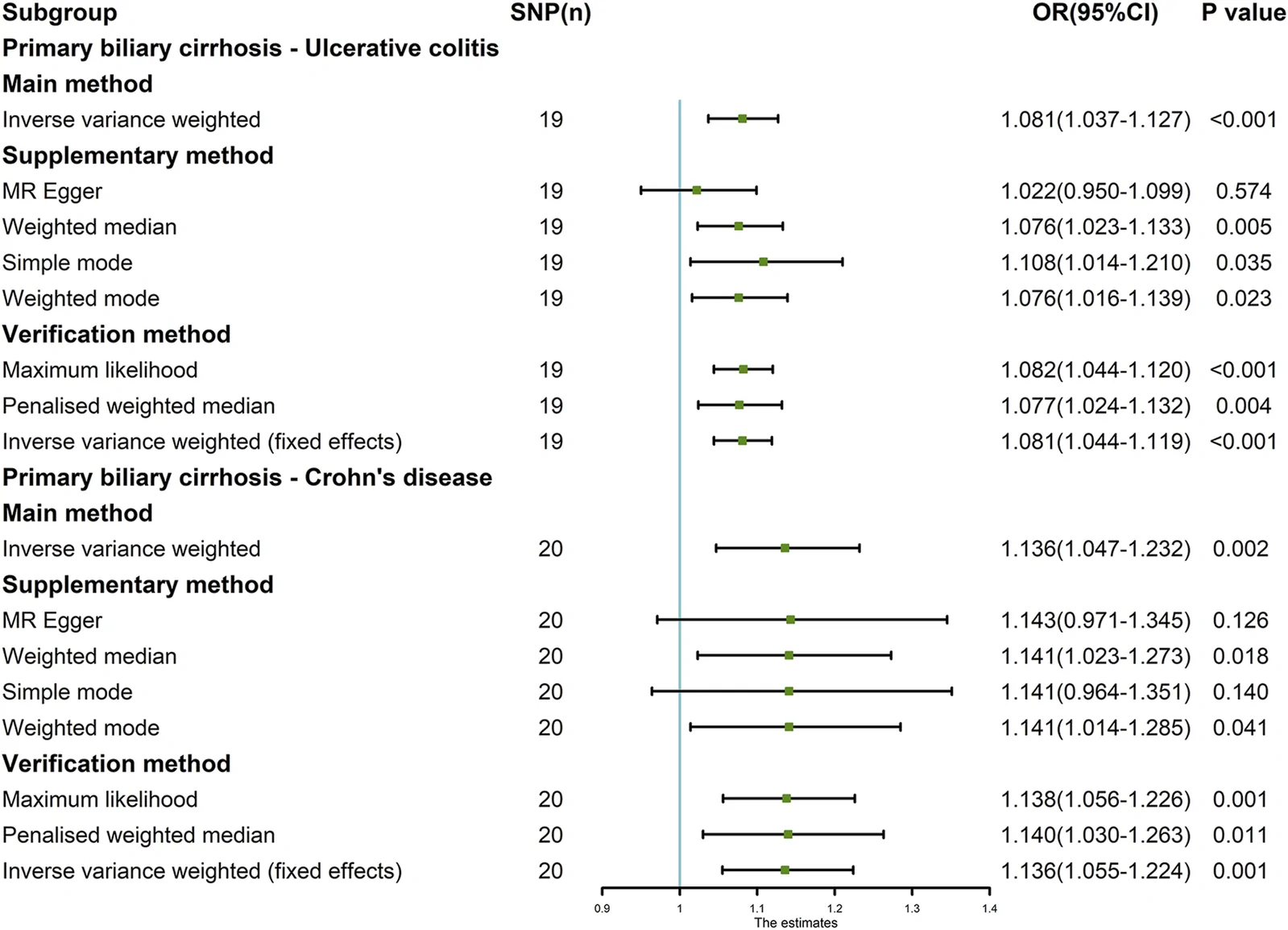
Forward MR analysis, primary biliary cirrhosis on ulcerative colitis and Crohn’s disease. This investigation employs a comprehensive suite of methodological approaches, encompassing random-effects IVW, MR Egger, weighted median, simple mode, weighted mode, maximum likelihood, penalized weighted median, and fixed-effects IVW methodologies.

**FIGURE 2:**
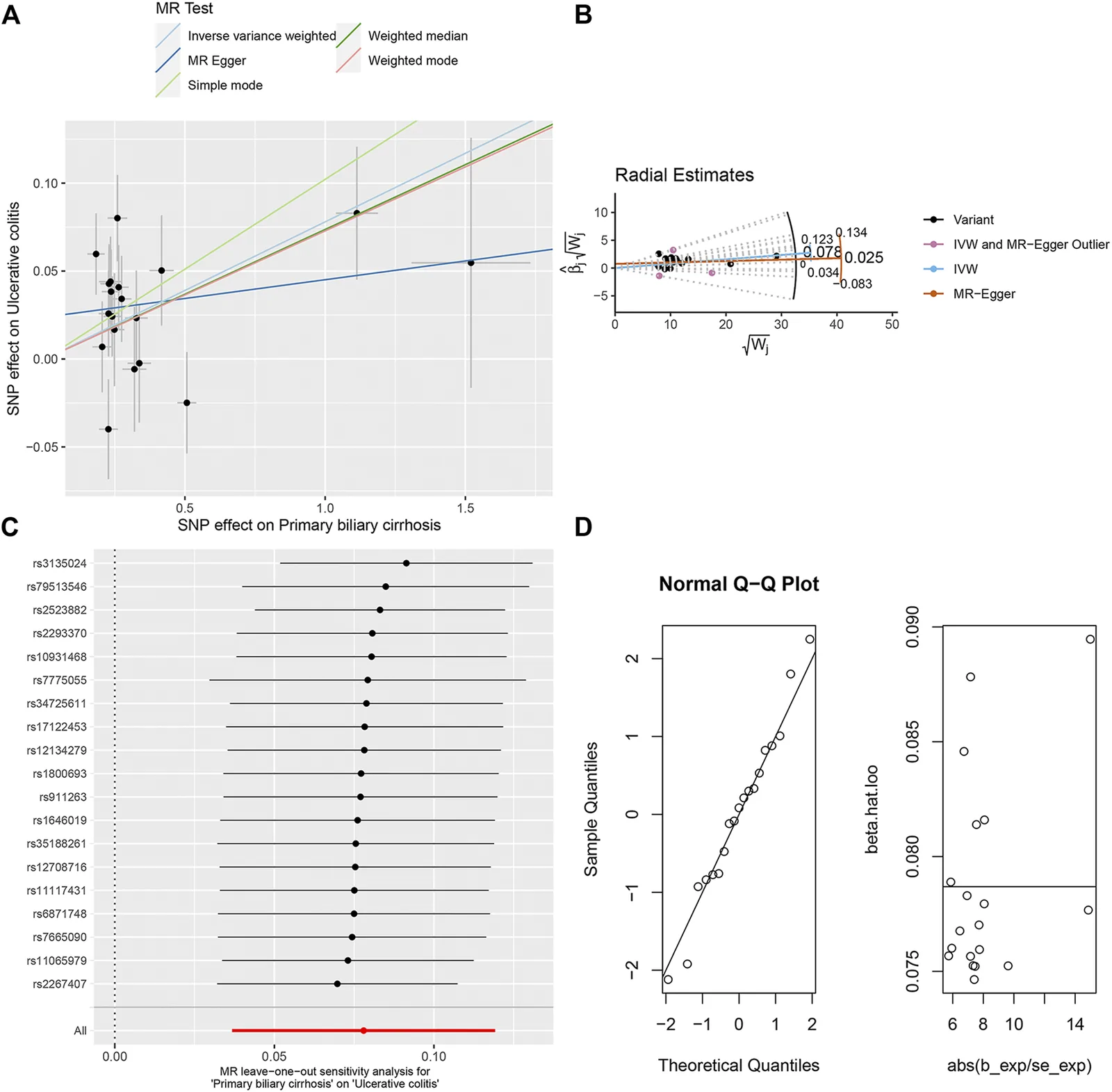
The forward MR analysis of primary biliary cirrhosis on ulcerative colitis. **(A)** Scatter plot; **(B)** MR radial plots; **(C)** Leave-one-out analysis; **(D)** Normal distribution.

**TABLE 1:** Sensitivity analysis of the MR analysis results of exposure and outcome.

Exposure	Outcome	Heterogeneity test		Pleiotropy test	MR-PRESSO		MR-RAPS
		Cochran’s Q test (IVW)	Rucker’s Q test (MR-Egger)	Egger Intercept (MR-Egger)	Distortion test (outliers)	Global Test (pleiotropy)	Normal Distribution
		P value	P value	P value	Number	P value	P value
PBC	UC	0.106	0.201	0.089	0	0.146	0.926
PBC	CD	0.253	0.205	0.936	0	0.308	0.482
UC	PBC	0.791	0.966	0.139	0	0.809	0.759
CD	PBC	0.784	0.994	0.412	0	0.787	—
PBC	IBD	0.511	0.463	0.561	0	0.499	0.437
IBD	PBC	0.069	0.086	0.318	0	0.076	0.516

MR, mendelian randomization; PBC, Primary biliary cirrhosis; UC, Ulcerative colitis; CD, Crohn’s disease; IBD, inflammatory bowel disease.

### Genetic causality between PBC on CD

After adjusting for LD, we identified 22 SNPs exhibiting strong correlation with PBC. All 22 SNPs are present in the GWAS summary data for CD. Notably, there were no palindromic SNPs among them. None of these 22 SNPs demonstrated an association with CD. It is crucial to mention that the confounding SNP rs11065979 was excluded from the analysis. Consequently, 21 SNPs were selected as IVs for the genetic causality assessment between PBC on CD (Supplementary Table S4).

Random-effects IVW analysis indicated a positive genetic causality between PBC on CD (P = 0.030, OR 95% CI = 1.114 [1.011–1.227]). Supplementary analyses using weighted median and weighted mode methods corroborated the random-effects IVW findings, whereas MR Egger and simple mode yielded inconsistent results (Supplementary Figures S1, S4A). Nonetheless, all three validation methods were in agree with the random-effects IVW results (Supplementary Figure S1). Cochran’s Q statistic for MR-IVW and Rucker’s Q statistic for MR Egger revealed significant heterogeneity (P < 0.05). Although MR Egger’s intercept test did not detect horizontal pleiotropy (P > 0.05), the global test of MR-PRESSO confirmed its presence (P < 0.05) (Supplementary Table S7). Outlier detection analyses identified outliers in the radial variants of IVW and MR Egger (Supplementary Figure S4B), and the MR-PRESSO distortion test also detected a significant outlier (rs10931468) (Supplementary Table S7). Leave-one-out analysis indicated that the genetic causal assessment was influenced by individual SNPs (rs79513546, rs35188261, rs7775055, rs2267407) (Supplementary Figure S4C). MR-RAPS analysis demonstrated that the genetic causal assessment was normally distributed (P > 0.05) (Supplementary Figure S4D; Supplementary Table S7).

Due to the inability of the sensitivity analysis to support the initial MR analysis results, a second round of MR analysis was conducted after excluding the outlier. Consistent with the initial findings, the random-effects IVW analysis continued to indicate a positive genetic causal relationship between PBC on CD (P = 0.002, OR 95% CI = 1.136 [1.047–1.232]). Despite the conflicting results from MR Egger and simple mode, the weighted median and weighted mode analyses supported the random-effects IVW findings (Figures 1, 3A). Furthermore, the three verification methods consistently indicated a positive genetic causal relationship between PBC and CD (Figure 1). No evidence of heterogeneity or horizontal pleiotropy was observed (P > 0.05) (Table 1). Although radial variants in IVW and MR Egger analyses identified outliers (Figure 3B), the MR-PRESSO distortion test did not detect any outliers (Table 1). The genetic causal assessment was not influenced by individual SNPs (Figure 3C) and adhered to a normal distribution (P > 0.05) (Figure 3D; Table 1).

**FIGURE 3:**
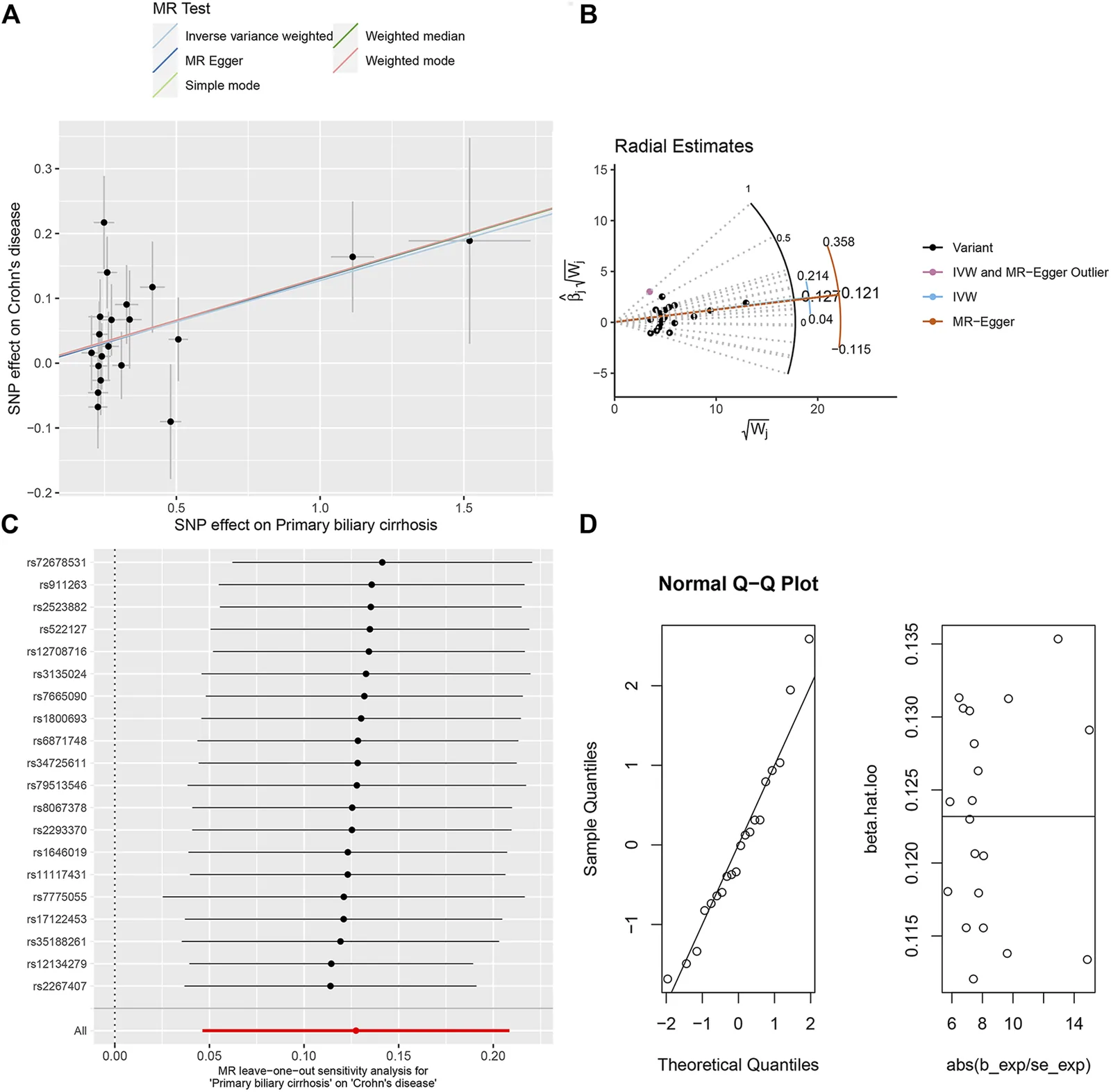
The forward MR analysis of primary biliary cirrhosis on Crohn’s disease. **(A)** Scatter plot; **(B)** MR radial plots; **(C)** Leave-one-out analysis; **(D)** Normal distribution.

### Genetic causality between UC on PBC

At a correlation threshold of P < 5 × 10^-8^, an insufficient number of SNPs were available for subsequent MR analysis. Consequently, we adjusted the correlation threshold to P < 5 × 10^-6^. This adjustment yielded 30 SNPs that are strongly associated with UC, 13 of which are present in the GWAS summary data for PBC. No palindromic SNPs were identified. One SNP associated with PBC, rs12946510, was excluded from the analysis. No confounding SNPs were detected. Ultimately, we identified 12 SNPs to serve as IVs for the genetic causality assessment of UC on PBC (Supplementary Table S5).

The random-effects IVW analysis revealed no evidence of genetic causality between UC on PBC (P = 0.575, OR 95% CI = 0.942 [0.763–1.162]). This result was corroborated by four supplementary methods (Supplementary Figures S5, S6A). Notably, the penalized weighted median analysis suggested a negative genetic causality between UC on PBC (Supplementary Figure S5). However, the sensitivity analysis indicated heterogeneity in the genetic causal assessment (P < 0.05). While MR Egger’s intercept test suggested the absence of horizontal pleiotropy (P > 0.05), the global test of MR-PRESSO provided evidence for horizontal pleiotropy (P < 0.05) (Supplementary Table S7). Radial variant analysis in both IVW and MR Egger revealed outliers in the genetic causal assessment (Supplementary Figure S6B), and the distortion test of MR-PRESSO identified an outlier (rs3024493) (Supplementary Table S7). The leave-one-out analysis did not identify any single SNPs influencing the outcome of the genetic causal assessment (Supplementary Figure S6C). MR-RAPS analysis indicated a normal distribution (P > 0.05) (Supplementary Figure S6D; Supplementary Table S7). Subsequent MR analysis, after removing an outlier, still demonstrated no genetic causal relationship between UC on PBC (P = 0.174, OR 95% CI = 0.882 [0.735–1.057]). Except MR Egger, this finding was supported by three other complementary methods (Supplementary Figures S5, S7A). Among these verification methods, the penalized weighted median was consistent with MR Egger, indicated a negative genetic causal relationship between UC on PBC. The maximum likelihood and fixed effects IV analyses were consistent with the random-effects IVW results (Supplementary Figure S5). No heterogeneity was observed in the sensitivity analysis (P > 0.05). The MR Egger intercept test indicated the presence of horizontal pleiotropy (P < 0.05), whereas the global test of MR-PRESSO did not detect horizontal pleiotropy (P > 0.05) (Supplementary Table S7). Radial variant analyses in both IVW and MR Egger demonstrated the presence of outliers (Supplementary Figure S7B), but the distortion test of MR-PRESSO did not identify outliers (Supplementary Table S7). Importantly, the leave-one- out analysis showed that two individual SNPs (rs7936070, rs3197999) influenced the genetic causal assessment (Supplementary Figure S7C). MR-RAPS continued to demonstrate that the genetic causal assessment conformed to a normal distribution (P > 0.05) (Supplementary Figure S7D; Supplementary Table S7).

Finally, a third round of MR analysis was conducted after excluding two SNPs that impacted the genetic causal assessment results. The random-effects IVW analysis revealed a negative genetic causality between UC on PBC (P = 0.003, OR 95% CI = 0.788 [0.672–0.924]). Among the four supplementary methods employed, MR-Egger and weighted median analyses supported this finding, whereas simple mode and weighted mode analyses suggested no genetic causal relationship between UC on PBC (Figures 4, 5A). Nevertheless, all three validation methods provided evidence of a negative genetic causality between UC on PBC (Figure 4). Sensitivity analyses did not detect heterogeneity or horizontal pleiotropy (P > 0.05) (Table 1). Furthermore, there was no evidence of outliers (Figure 5B; Table 1). The genetic causality assessment remained robust when individual SNPs were considered (Figure 5C), and the results were normally distributed (P > 0.05) (Figure 5D; Table 1).

**FIGURE 4:**
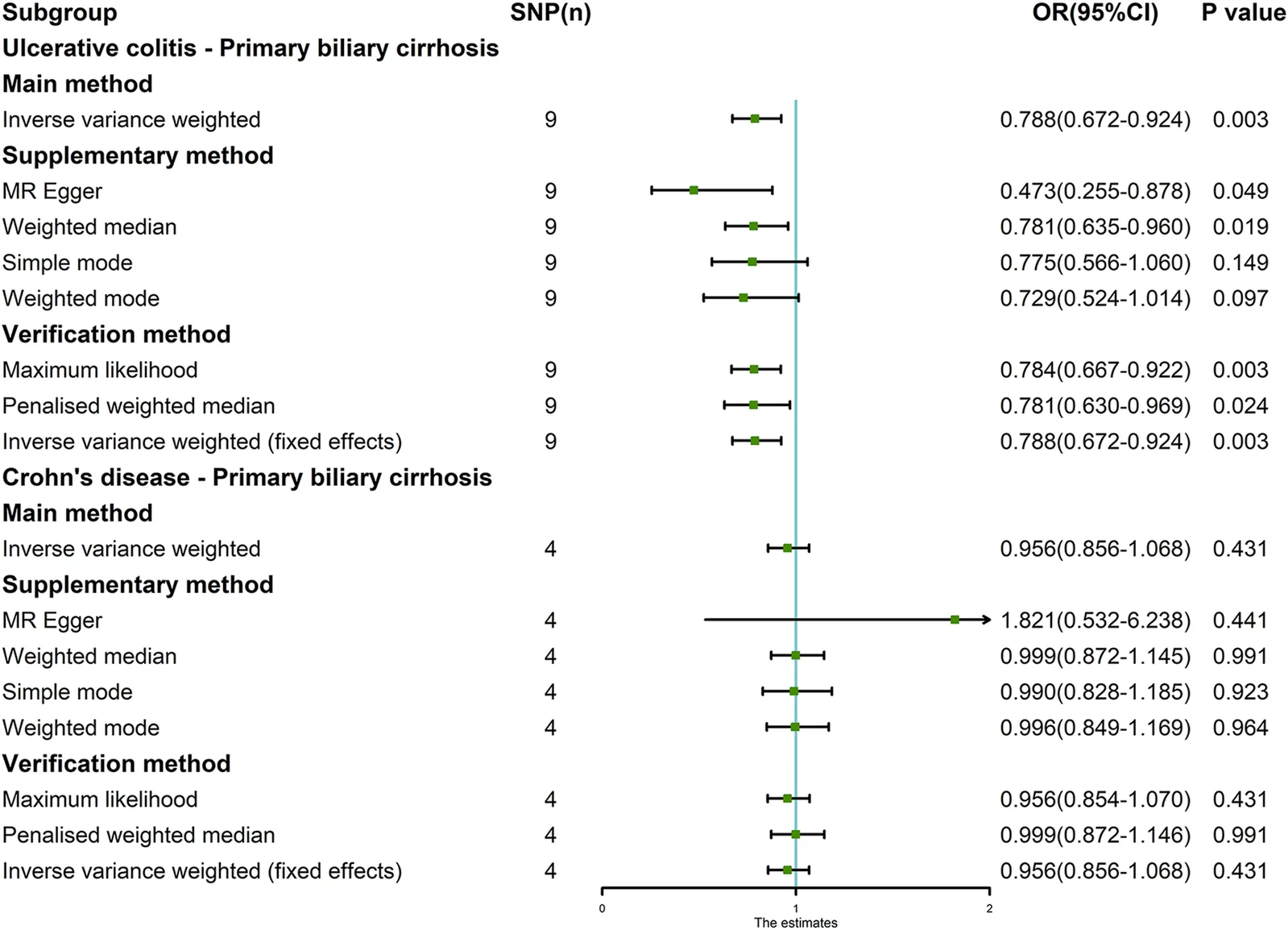
Reverse MR analysis, ulcerative colitis and Crohn’s disease on primary biliary cirrhosis. This investigation employs a comprehensive suite of methodological approaches, encompassing random-effects IVW, MR Egger, weighted median, simple mode, weighted mode, maximum likelihood, penalized weighted median, and fixed-effects IVW methodologies.

**FIGURE 5:**
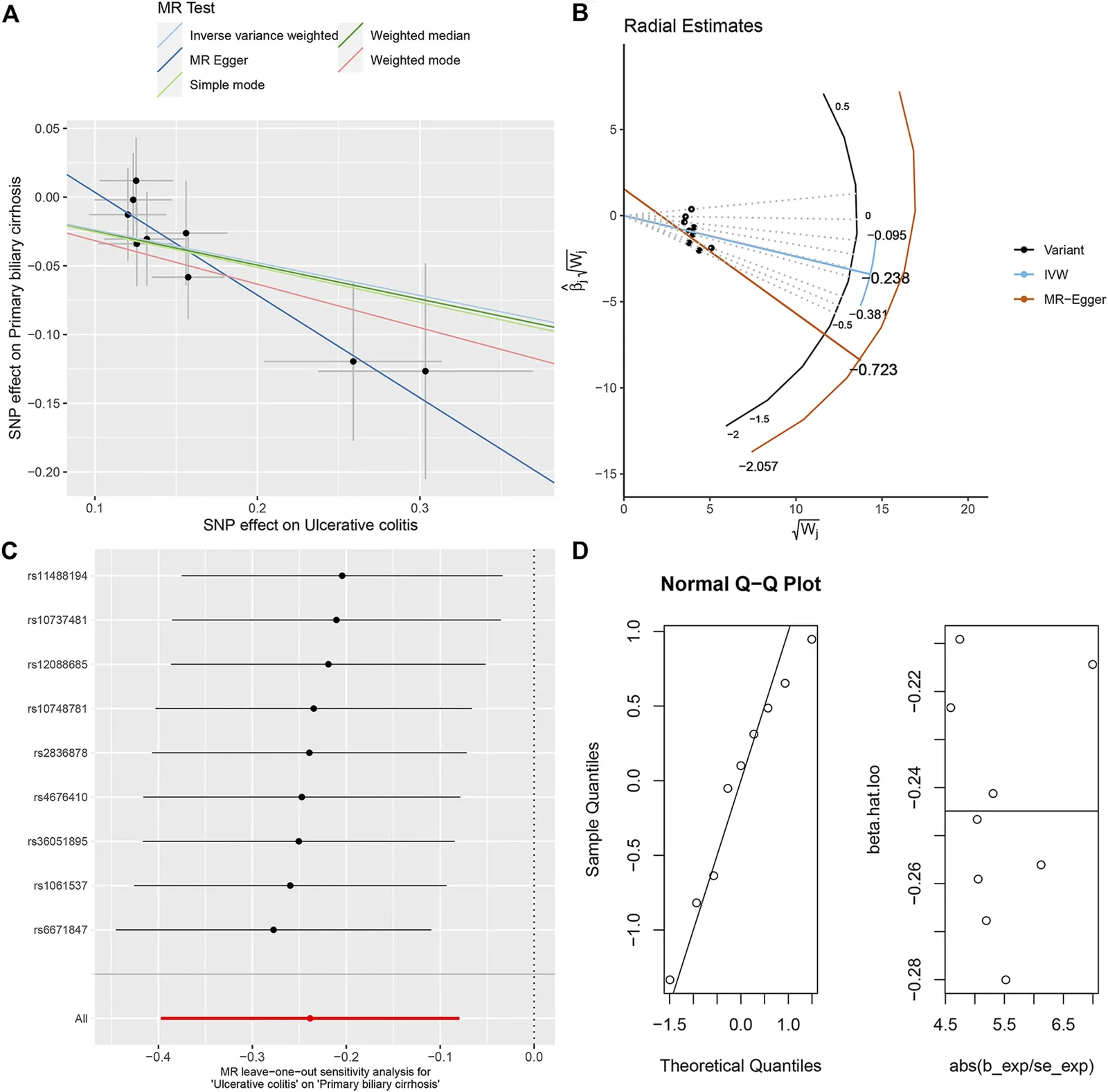
The forward MR analysis of ulcerative colitis on primary biliary cirrhosis. **(A)** Scatter plot; **(B)** MR radial plots; **(C)** Leave-one-out analysis; **(D)** Normal distribution.

### Genetic causality between CD on PBC

Under the initial correlation threshold of P < 5 × 10^-8^, it was not possible to obtain sufficient SNPs for subsequent MR analysis. Therefore, the correlation threshold was adjusted to P < 5 × 10^-6^, resulting in the identification of 22 SNPs strongly associated with CD, of which 4 SNPs were also present in the GWAS summary data for PBC. Notably, there were no palindromic SNPs, no SNPs related to PBC, and no confounding SNPs identified. Consequently, these 4 SNPs were utilized as IVs for the genetic causal assessment of CD on PBC (Supplementary Table S6).

The random-effects IVW analysis indicated no genetic causal relationship between CD on PBC (P = 0.431, OR 95% CI = 0.956 [0.856–1.068]). Four supplementary methods corroborated the findings of the random-effects IVW analysis (Figures 4, 6A). Similarly, three verification methods produced consistent results (Figure 4). Both Cochran’s Q statistic for MR-IVW and Rucker’s Q statistic for MR Egger indicated no heterogeneity in the genetic causal assessment of CD on PBC (P > 0.05). Moreover, the intercept test of MR Egger and the global test of MR-PRESSO did not reveal the presence of horizontal pleiotropy (P > 0.05) (Table 1). Due to the limited number of IVs, radial variants of IVW and MR Egger are not illustrated. The MR-PRESSO distortion test did not detect any outliers (Table 1). Leave-one-out analysis demonstrated that the genetic causality assessment was robust to the retention of individual SNPs (Figure 6B). Additionally, MR-RAPS analysis indicated a normal distribution (P > 0.05) (Figure 6C; Table 1).

**FIGURE 6:**
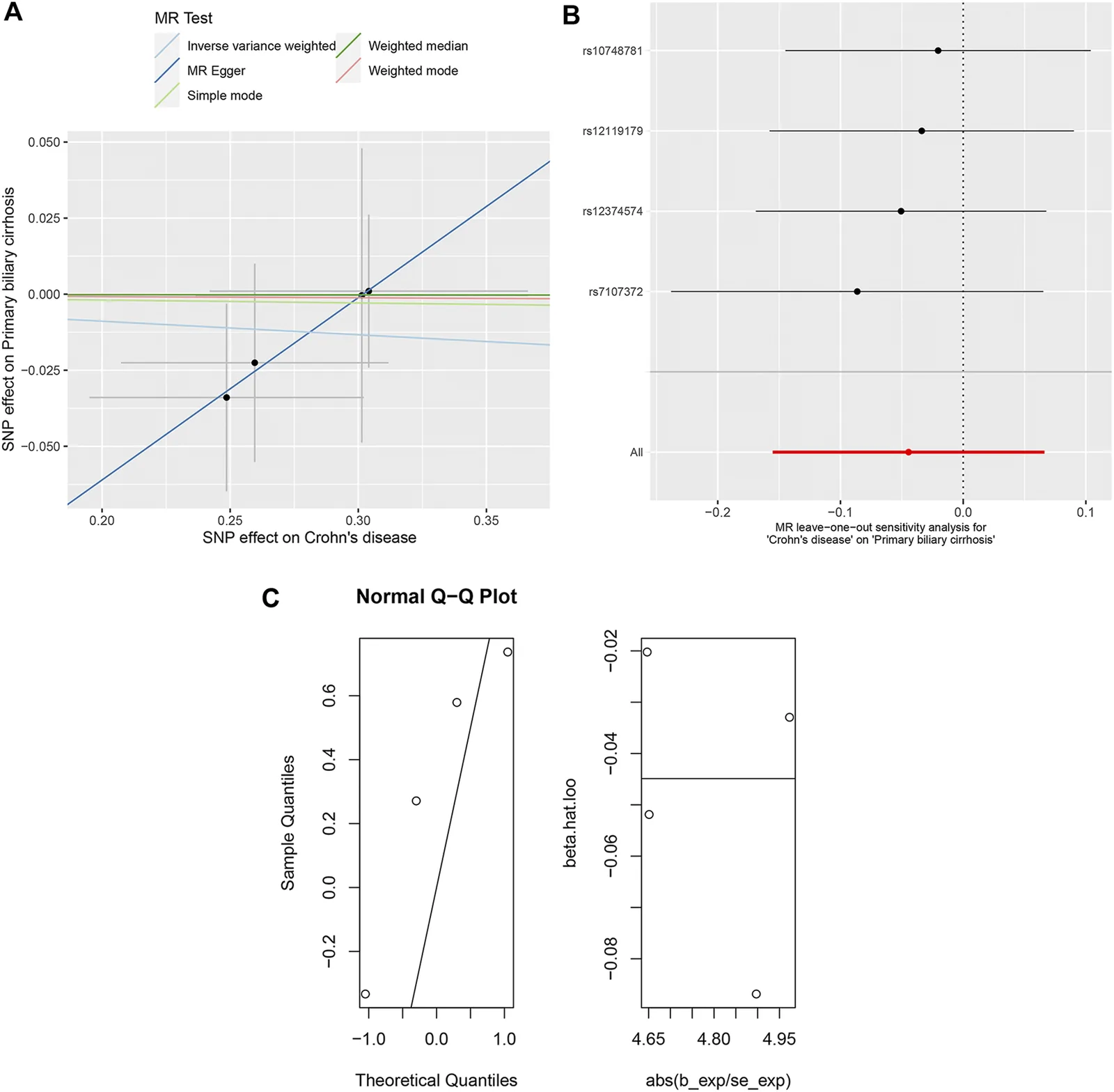
The forward MR analysis of Crohn’s disease on primary biliary cirrhosis. (A) Scatter plot; (B) Leave-one-out analysis; (C) Normal distribution.

### Genetic causality between PBC and IBD

Following LD pruning, we first identified 22 SNPs that were strongly associated with PBC. All of these variants were successfully retrieved from the IBD GWAS summary data, and none were palindromic. Subsequent checks confirmed that none of the 22 SNPs exhibited an independent association with IBD. We then proceeded to exclude one confounding SNP (rs11065979) and to remove four outlier variants (rs2267407, rs522127, rs72678531, rs8067378) in advance. After these quality-control steps, a final set of 17 SNPs was retained as IVs for estimating the causal effect of PBC on IBD (Supplementary Table S8). For the reverse direction, LD pruning yielded 9 SNPs that were strongly correlated with IBD. These variants were all available in the PBC GWAS summary statistics, with no palindromic or confounding SNPs detected, and none showed any association with PBC. Accordingly, all 9 SNPs were directly adopted as IVs for assessing the causal impact of IBD on PBC (Supplementary Table S9).

The random-effects IVW method revealed a significant positive genetic causal relationship of PBC on IBD (P < 0.001, OR 95% CI = 1.076 [1.042–1.110]). This finding was consistently supported by three supplementary methods and further corroborated by three additional verification approaches (Figures 7, 8A). Conversely, the reverse-direction analysis, using the same random-effects IVW model, showed no genetic causal effect of IBD on PBC (P = 0.357, OR 95% CI = 0.898 [0.714–1.129]). This null result was likewise confirmed by four supplementary methods and three verification procedures (Figures 7, 8C). Sensitivity analyses indicated the absence of heterogeneity, horizontal pleiotropy, and outlier variants, with the residuals conforming to a normal distribution (Table 1). Furthermore, leave-one-out analysis demonstrated that no single SNP disproportionately influenced the causal estimates (Figures 8B,D).

**FIGURE 7:**
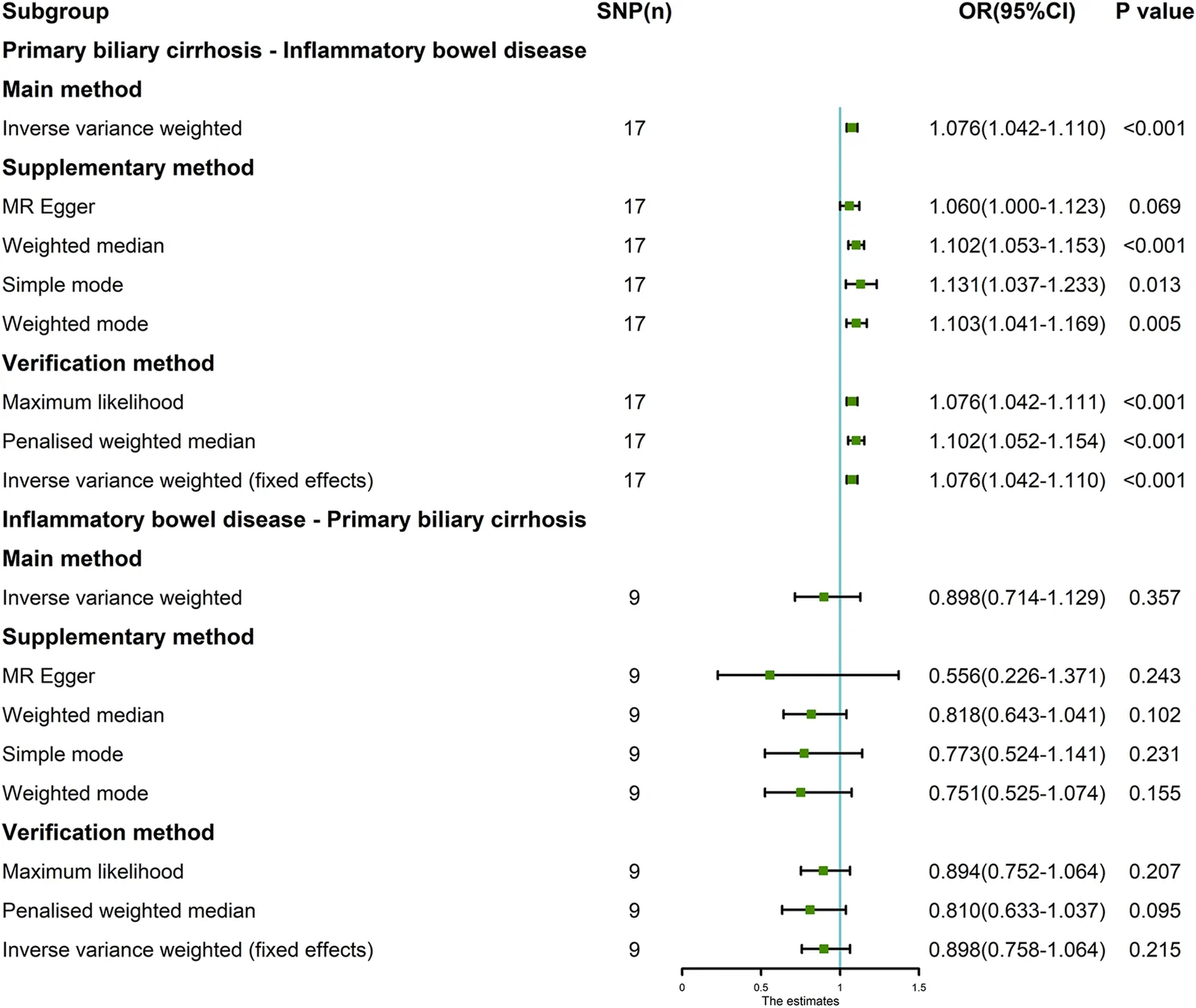
Bidirectional MR analysis between primary biliary cirrhosis and inflammatory bowel disease. This investigation employs a comprehensive suite of methodological approaches, encompassing random-effects IVW, MR Egger, weighted median, simple mode, weighted mode, maximum likelihood, penalized weighted median, and fixed-effects IVW methodologies.

**FIGURE 8:**
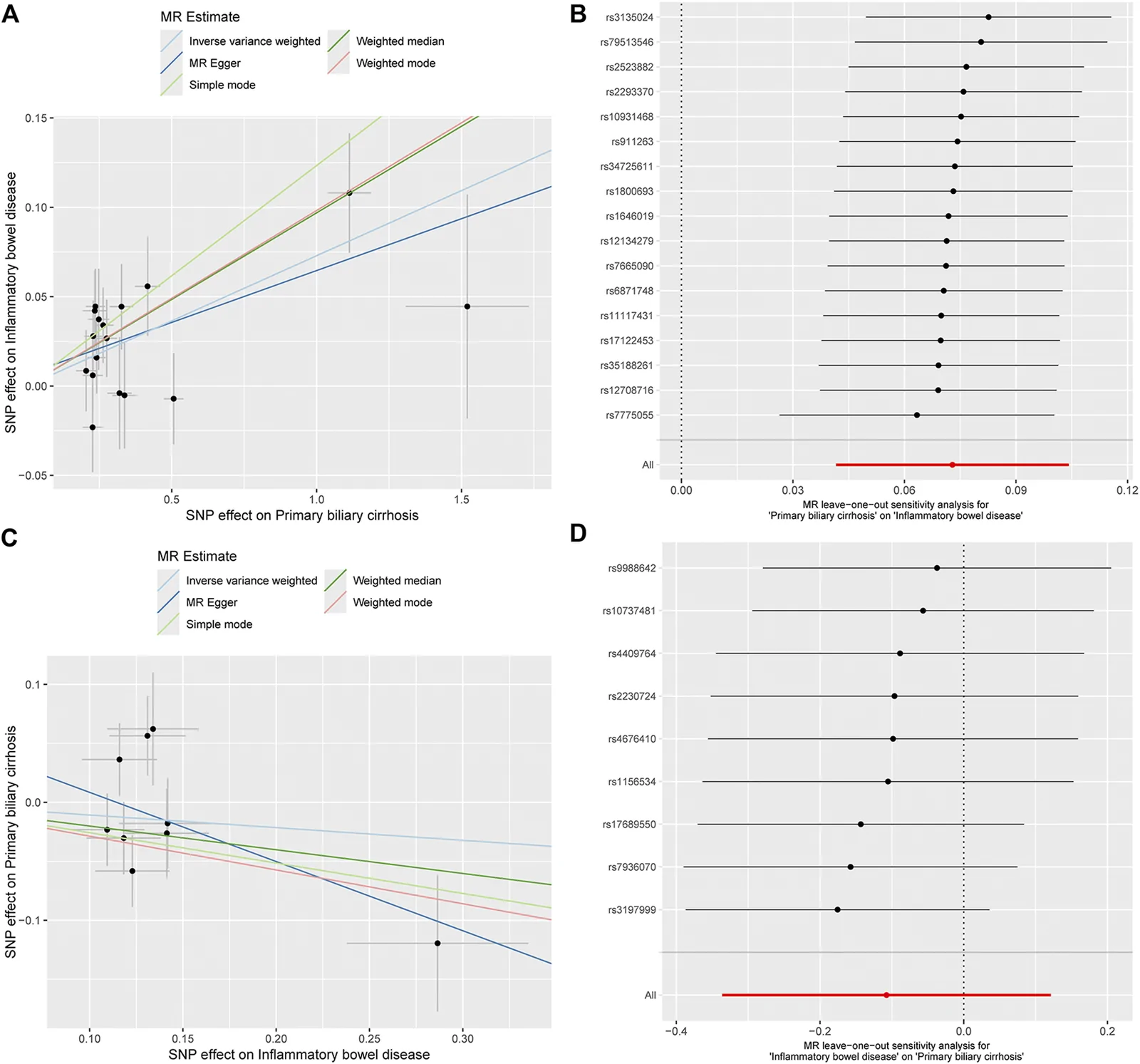
Bidirectional MR analysis between primary biliary cirrhosis and inflammatory bowel disease. **(A)** Scatter plot of primary biliary cirrhosis on inflammatory bowel disease; **(B)** Leave-one-out analysis of primary biliary cirrhosis on inflammatory bowel disease; **(C)** Scatter plot of inflammatory bowel disease on primary biliary cirrhosis; **(D)** Leave-one-out analysis of inflammatory bowel disease on primary biliary cirrhosis.

## Discussion

In this study, we performed a bidirectional two-sample MR analysis to systematically evaluate the genetic causal effects of PBC on IBD, and *vice versa*. The forward MR analysis revealed significant positive causal effects of PBC on both UC and CD, indicating that PBC serves as a genetic predisposing factor for each IBD subtype. In the reverse direction, however, we observed a negative causal effect of UC on PBC—suggesting a protective genetic role—whereas no such effect was detected for CD on PBC. When IBD was considered as a composite phenotype, the positive causal effect of PBC on overall IBD remained consistent, while the effect of IBD on PBC was not supported. Collectively, these findings delineate a directional and subtype-specific genetic causality between PBC and IBD.

UC exhibits a notable correlation with a spectrum of hepatic and biliary disorders, encompassing PBC, PSC, hepatic steatosis, and amyloidosis. The prevalence of persistent aberrations in liver function tests among UC patients ranges from 3% to 15%, while autopsy or surgical findings indicate abnormal liver histology in up to 90% of cases [35]. The precise nature of the interplay between UC and PBC remains enigmatic, although conjectures regarding genetic and immunological influences have been posited. Genetic predisposition significantly contributes to the pathogenesis of both conditions, particularly evidenced by the association of UC and PBC with genetic loci on the short arm of chromosome 6, notably within the HLA class II region, implicating regulatory genes pivotal in inflammation-related pathogenesis [36]. The involvement of the immune system in the pathogenesis of UC-associated autoimmune hepatobiliary diseases such as PBC is evidenced by lymphocyte infiltration in the portal vein and the presence of circulating immune complexes and antibodies reactive with the bile duct [37]. In Europe, the prevalence of PBC is approximately 15 per 100,000 individuals. Among 412 UC cohorts, two cases of PBC were diagnosed, yielding an estimated prevalence of PBC in these patients at 485 per 100,000, nearly 30 times higher than that of the general population [38]. Notably, of the 14 patients with PBC, 13 had been previously diagnosed with UC, typically many years earlier. The onset of PBC symptoms commonly coincides with the active phase of UC, suggesting a linkage wherein the progression of PBC appears contingent upon UC activity. Consequently, PBC is widely regarded as an extraintestinal (hepatobiliary) manifestation of UC. Furthermore, investigations have identified instances where patients initially diagnosed with PBC later developed UC, thereby suggesting UC as an extrahepatic (intestinal) manifestation of PBC [35].

*Helicobacter pylori* has been implicated in liver disease, with genetic sequences of *Helicobacter pylori* species detected in liver tissue samples from PBC patients [39]. PCR analysis utilizing *Helicobacter* pylori-specific primers revealed positivity in 20 out of 24 liver samples from patients with PSC or PBC, with nine samples specifically testing positive for *Helicobacter pylori* by PCR(39). *In vitro* experimentation has demonstrated the sensitivity of *Helicobacter pylori* to major free cholic acid, deoxycholic acid, and chemical deoxycholic acid present in human bile. Notably, in *Helicobacter* pylori-positive patients, elevated alkaline phosphatase (ALP) and protein tyrosine kinase (PTK) levels were observed, underscoring a significant association with biliary sedimentary liver disease [39]. In summary, the coexistence of UC and PBC is evident, with cases presenting either UC or PBC as the initial diagnosis. While a genetic and immune association between UC and PBC is established, the causal relationship between the two remains elusive. This study contributes to elucidating the genetic underpinnings of the relationship between UC and PBC, suggesting PBC as a genetic predisposing factor for UC, and conversely, UC as a genetic protective factor for PBC. This intriguing discovery addresses a lacuna in the existing literature, prompting the necessity for further investigation to elucidate and expand upon this inquiry in subsequent studies.

CD has been documented as an extrahepatobiliary manifestation of PBC, although instances of PBC co-occurring with CD have been reported [40]. Despite previous investigations, the precise interrelation between PBC and CD has remained ambiguous. This present study elucidates a causal nexus between the two conditions at the genetic level, suggesting PBC as a genetic predisposing factor for CD. IBD comprise a cluster of autoimmune disorders characterized by unresolved gastrointestinal inflammation. Genome-wide scrutiny has identified shared autophagy-related genes implicated in IBD, notably ATG16L1, IRGM, and LRRK2 [41]. Functional exploration of CD-associated genetic variants within ATG16L1 and IRGM has further unveiled impairment in mitochondrial autophagy, evidenced by morphological alterations in mitochondria, diminished mitochondrial membrane potential, escalated mitochondrial ROS (mtROS), and perturbed LC3-mediated autophagy [42]. Pathologically, PBC encompasses chronic progressive destruction of small bile ducts (SBD), portal vein inflammation, and eventual cirrhosis [43]. The aberrant SBD damage in PBC stems from an abnormal immune reaction targeting mitochondrial autoantigens. Notably, Dihydrolipoamide S-acetyltransferase (DLAT/PDC-E2) and oxoglutarate dehydrogenase (OGDH) complex serve as primary mitochondrial autoantigens (mtAg), eliciting augmented responses from CD4^+^ and CD8^+^ T cells in PBC [43, 44]. In essence, the correlation between PBC and CD may hinge upon mitochondrial autophagy. A study conducted on a Japanese populace identified shared susceptibility genes for PBC and CD, encompassing CXCR5, ICOSLG, STAT4, IL12B, NFKB1, and TNFSF15. Notably, TNFSF15, CXCR5, and ICOSLG risk alleles exhibited analogous effects on disease susceptibility, implying a potential shared pathogenic pathway in PBC and CD development [19]. Conversely, risk alleles pertaining to IL12B, STAT4, and NFKB1 displayed inverse associations in both maladies, hinting at divergent modulation of Th1 and Th17 polarization through the IL12-STAT4-NFKB signaling axis [19]. These findings offer insights into the pathogenesis of PBC and CD. Additionally, TNFSF15-encoded TL1A involvement in apoptosis and immune responses, fostering Th1 and Th17 differentiation, underscores its potential pivotal role in the pathogenesis of both diseases through these mechanisms [45].

Despite methodological rigour, this study has two principal limitations. First, the use of European-only GWAS data, while minimising population stratification, restricts the generalisability of our findings to other ethnic groups, as allele frequencies, linkage disequilibrium, gene–environment interactions, and phenotypic heterogeneity may differ substantially across ancestries. Future work should incorporate multi-ancestry GWAS data (e.g., trans-ethnic meta-analyses) and validate results using polygenic risk scores in independent non-European cohorts to assess cross-population stability. Second, the number of instrumental variables is relatively small due to stringent selection criteria (genome-wide significance, LD clumping, F-statistic >10), which may reduce statistical power to detect modest effects and limit coverage of the exposure’s genetic architecture. Nevertheless, our sensitivity analyses (MR-Egger, weighted median, etc.) support the robustness of the findings. To address this, subsequent studies could expand IV sets through larger GWAS samples, integrate multi-omics data (eQTL/pQTL), apply multivariate MR frameworks to improve precision, and perform replication in external independent GWAS datasets. In summary, while these limitations are acknowledged, our core conclusions remain robust, and future efforts combining larger, multi-ancestry, and multi-omics MR designs will further solidify and extend our causal inferences for clinical and public health translation.

## Conclusion

In this study, we employed a bidirectional two-sample MR framework to elucidate the genetic causal effects of PBC and IBD. The forward MR analysis provided compelling evidence of positive genetic effects of PBC on both UC and CD, establishing PBC as a shared genetic predisposing factor for both IBD subtypes. This finding carries clinical relevance: individuals diagnosed with PBC may warrant heightened vigilance and potentially routine screening for incident UC and CD. In the reverse direction, we observed a significant negative genetic effect of UC on PBC—suggesting a protective role—whereas no such effect was detected for CD on PBC. When IBD was considered as a composite phenotype, the positive effect of PBC on overall IBD remained consistent, while the effect of IBD on PBC was not supported. Collectively, these results delineate a directional and subtype-specific genetic causality between PBC and IBD, with implications for risk stratification and clinical surveillance.

## Data Availability

Publicly available datasets were analyzed in this study. This data can be found here: https://opengwas.io/.
