# Supplementary material for: Primary biliary cirrhosis and inflammatory bowel disease: a two-sample bidirectional Mendelian randomization study
Source: Exp Biol Med (Maywood). 2026 Sep 17;251:10940. doi: 10.3389/ebm.2026.10940 (PMC13627073; doi:10.3389/ebm.2026.10940)
Supplement: Supplementary file 1 [file Table1.doc]

**Supplementary Table 1:** STROBE-MR checklist of MR study.

| **Item No** | **Section** | **Checklist item** |
| --- | --- | --- |
| 1 | **Title** | Primary biliary cirrhosis and inflammatory bowel disease: A two-sample bidirectional Mendelian randomization study |
| 2 | **Abstract** | Observational studies have frequently reported an association between primary biliary cirrhosis (PBC) and inflammatory bowel disease (IBD). In this study, we leveraged summary‑level data from genome‑wide association studies (GWAS) to conduct a two‑sample bidirectional Mendelian randomization (MR) analysis, with the primary objective of investigating the genetic causal relationship between PBC and IBD, comprising ulcerative colitis (UC) and Crohn's disease (CD). Additionally, a validation analysis was performed by repeating the bidirectional MR framework, alternately defining PBC and IBD as the exposure and outcome variables to confirm the directionality of the observed associations. A comprehensive panel of sensitivity analyses was implemented to test the robustness of the findings. We first examined the genetic causality at the subtype level. In the forward MR, PBC exerted a significant positive causal effect on UC (P < 0.001, OR 95% CI = 1.081 [1.037-1.127]) and on CD (P = 0.002, OR 95% CI = 1.136 [1.047-1.232]). In the reverse MR, only UC showed a significant negative causal influence on PBC (P > 0.003, OR 95% CI = 0.788 [0.672-0.924]), whereas no significant effect was detected from CD on PBC (P = 0.431, OR 95% CI = 0.956 [0.856-1.068]). We then extended the analysis to the overall IBD phenotype. The forward MR demonstrated a significant positive genetic relationship between PBC on IBD (P < 0.001, OR 95% CI = 1.076 [1.042-1.110]). Conversely, the reverse MR did not support a causal effect of IBD on PBC (P = 0.357, OR 95% CI = 0.898 [0.714-1.129]). The robustness of all these findings was confirmed by comprehensive sensitivity analyses, which showed no evidence of heterogeneity, horizontal pleiotropy, or undue influence from individual instrumental variables. Our MR analysis demonstrates that PBC serves as a genetic determinant of IBD as a whole and of UC/CD separately, whereas reverse causation is limited to a protective effect of UC on PBC. This direction‑dependent and subtype‑specific causal architecture provides novel insights into the shared etiological pathways between PBC and IBD. |
|  | **Introduction** |  |
| 3 | Background | Inflammatory bowel disease (IBD), encompassing ulcerative colitis (UC) and Crohn's disease (CD), is characterized by chronic, progressive immune-mediated inflammation of the gastrointestinal tract. Primary biliary cirrhosis (PBC) is an autoimmune liver disease characterized by the presence of highly specific anti-mitochondrial antibodies (AMAs) and autoreactive T cells in the serum. Previous studies have demonstrated a close relationship between PBC and IBD, suggesting mutual influences. The reduced bile flow resulting from PBC’s immune-mediated tissue damage and the accumulation of toxic bile products not only perpetuate biliary epithelial damage but also alter the composition of the gut and biliary microbiota and their interactions with the host. The polyclonal high IgM response and autoantibodies in PBC consistently cross-react with microbial antigens in IBD, leading to changes in the composition of the gut or biliary tract microbiota and subsequent alterations in epithelial integrity, which promote microbial translocation. However, the causal relationship between IBD and PBC remains unclear. Investigating this causal relationship is of significant importance for clinical prevention and treatment strategies. |
| 4 | Objectives | In this study, we conducted a two-sample bidirectional MR analysis utilizing aggregated summary data from an openly accessible GWAS to elucidate the genetic causality between PBC and IBD. |
|  | **Methods** |  |
| 5 | Study design | We conducted a two-sample bidirectional MR analysis to explore the genetic underpinnings of the causal relationship between PBC and IBD. Initially, a forward MR analysis was executed, wherein PBC served as the exposure variable, while UC and CD were considered as outcome variables. Subsequently, a reverse MR analysis was undertaken, with UC and CD as exposure variables and PBC as the outcome variable. The validation analysis conducted a bidirectional MR analysis, treating PBC and IBD as the exposure and outcome variables respectively. |
| 6 | Assumptions |  |
|  | a) | The IVs are strongly correlated with the exposure variables. |
|  | b) | The IVs are not associated with the outcome variables or confounding factors. |
|  | c) | The IVs solely influence outcomes through the exposure. |
| 7 | Data sources |  |
|  | a) | The GWAS summary data for PBC were retrieved from the IEU OpenGWAS database (<https://gwas.mrcieu.ac.uk/>). |
|  | b) | The GWAS summary data for UC, CD and IBD were sourced from the Finnish consortium (<https://www.finngen.fi/>). |
| 8 | MR analysis |  |
|  | a) | Main method: random-effects IVW. |
|  | b) | Supplementary methods: MR Egger, Weighted median, Simple mode, and Weighted mode. |
|  | c) | Verification methods: Maximum likelihood, Penalised weighted median, and IVW (fixed effects). |
| 9 | Sensitivity analysis |  |
|  | a) | Heterogeneity: Cochran’s Q statistic for MR-IVW analyses and Rucker’s Q statistic for MR Egger analyses. |
|  | b) | Horizontal pleiotropy: intercept test of MR Egger and global test of MR-PRESSO. |
|  | c) | Outliers: distortion test of MR-PRESSO. |
|  | d) | Leave one out: detect whether the causal relationship was influenced by a single SNP. |
|  | e) | Normal distribution: MR-RAPS. |
| 10 | Software | R software (version 4.1.2) |
|  | **Results** |  |
| 11 | Descriptive data |  |
|  | a) | PBC on UC: 22 SNPs were used as IVs. |
|  | b) | PBC on CD: 21 SNPs were used as IVs. |
|  | c) | UC on PBC: 12 SNPs were used as IVs. |
|  | d) | CD on PBC: 4 SNPs were used as IVs. |
|  | e) | PBC on IBD: 17 SNPs were used as IVs. |
|  | f) | IBD on PBC: 9 SNPs were used as IVs. |
| 12 | Main results |  |
|  | a) | PBC and UC: positive genetic causal relationship. |
|  | b) | PBC and CD: positive genetic causal relationship. |
|  | c) | UC and PBC: negative genetic causal relationship. |
|  | d) | CD and PBC: no genetic causal relationship. |
|  | e) | PBC on IBD: positive genetic causal relationship. |
|  | f) | IBD on PBC: no genetic causal relationship. |
| 13 | Sensitivity analysis |  |
|  | a) | No heterogeneity. |
|  | b) | No horizontal pleiotropy. |
|  | c) | No outliers. |
|  | d) | The "leave one out" analysis indicated that the MR analysis results were not driven by a single SNP. |
|  | e | The MR analysis followed a normal distribution. |
|  | **Discussion** |  |
| 14 | Key results | In this study, we performed a bidirectional two‑sample MR analysis to systematically evaluate the genetic causal effects of PBC on IBD, and vice versa. The forward MR analysis revealed significant positive causal effects of PBC on both UC and CD, indicating that PBC serves as a genetic predisposing factor for each IBD subtype. In the reverse direction, however, we observed a negative causal effect of UC on PBC—suggesting a protective genetic role—whereas no such effect was detected for CD on PBC. When IBD was considered as a composite phenotype, the positive causal effect of PBC on overall IBD remained consistent, while the effect of IBD on PBC was not supported. Collectively, these findings delineate a directional and subtype‑specific genetic causality between PBC and IBD. |
| 15 | Interpretation |  |
|  | a) | UC exhibits a notable correlation with a spectrum of hepatic and biliary disorders, encompassing PBC, PSC, hepatic steatosis, and amyloidosis. The prevalence of persistent aberrations in liver function tests among UC patients ranges from 3% to 15%, while autopsy or surgical findings indicate abnormal liver histology in up to 90% of cases. The precise nature of the interplay between UC and PBC remains enigmatic, although conjectures regarding genetic and immunological influences have been posited. Genetic predisposition significantly contributes to the pathogenesis of both conditions, particularly evidenced by the association of UC and PBC with genetic loci on the short arm of chromosome 6, notably within the HLA class II region, implicating regulatory genes pivotal in inflammation-related pathogenesis. The involvement of the immune system in the pathogenesis of UC-associated autoimmune hepatobiliary diseases such as PBC is evidenced by lymphocyte infiltration in the portal vein and the presence of circulating immune complexes and antibodies reactive with the bile duct. |
|  | b) | Helicobacter pylori has been implicated in liver disease, with genetic sequences of Helicobacter pylori species detected in liver tissue samples from PBC patients. PCR analysis utilizing Helicobacter pylori-specific primers revealed positivity in 20 out of 24 liver samples from patients with PSC or PBC, with nine samples specifically testing positive for Helicobacter pylori by PCR. In vitro experimentation has demonstrated the sensitivity of Helicobacter pylori to major free cholic acid, deoxycholic acid, and chemical deoxycholic acid present in human bile. Notably, in Helicobacter pylori-positive patients, elevated alkaline phosphatase (ALP) and protein tyrosine kinase (PTK) levels were observed, underscoring a significant association with biliary sedimentary liver disease. In summary, the coexistence of UC and PBC is evident, with cases presenting either UC or PBC as the initial diagnosis. While a genetic and immune association between UC and PBC is established, the causal relationship between the two remains elusive. This study contributes to elucidating the genetic underpinnings of the relationship between UC and PBC, suggesting PBC as a genetic predisposing factor for UC, and conversely, UC as a genetic protective factor for PBC. This intriguing discovery addresses a lacuna in the existing literature, prompting the necessity for further investigation to elucidate and expand upon this inquiry in subsequent studies. |
|  | c) | CD has been documented as an extrahepatobiliary manifestation of PBC, although instances of PBC co-occurring with CD have been reported. Genome-wide scrutiny has identified shared autophagy-related genes implicated in IBD, notably ATG16L1, IRGM, and LRRK2. Functional exploration of CD-associated genetic variants within ATG16L1 and IRGM has further unveiled impairment in mitochondrial autophagy, evidenced by morphological alterations in mitochondria, diminished mitochondrial membrane potential, escalated mitochondrial ROS (mtROS), and perturbed LC3-mediated autophagy. In PBC patients, DLAT colocalizes with autophagy markers such as LC3 and SQSTM1 within damaged SBD. Subsequent in vitro investigations corroborated these findings in cultured bile epithelial cells, associating autophagy induction with heightened DLAT expression on the cell surface. In essence, the correlation between PBC and CD may hinge upon mitochondrial autophagy. Despite previous investigations, the precise interrelation between PBC and CD has remained ambiguous. This present study elucidates a causal nexus between the two conditions at the genetic level, suggesting PBC as a genetic predisposing factor for CD. |
| 16 | Limitations |  |
|  | a) | Despite methodological rigour, this study has two principal limitations. First, the use of European‑only GWAS data, while minimising population stratification, restricts the generalisability of our findings to other ethnic groups, as allele frequencies, linkage disequilibrium, gene–environment interactions, and phenotypic heterogeneity may differ substantially across ancestries. Future work should incorporate multi‑ancestry GWAS data (e.g., trans‑ethnic meta‑analyses) and validate results using polygenic risk scores in independent non‑European cohorts to assess cross‑population stability. |
|  | b) | Second, the number of instrumental variables is relatively small due to stringent selection criteria (genome‑wide significance, LD clumping, F‑statistic >10), which may reduce statistical power to detect modest effects and limit coverage of the exposure’s genetic architecture. Nevertheless, our sensitivity analyses (MR‑Egger, weighted median, etc.) support the robustness of the findings. To address this, subsequent studies could expand IV sets through larger GWAS samples, integrate multi‑omics data (eQTL/pQTL), apply multivariate MR frameworks to improve precision, and perform replication in external independent GWAS datasets. |
| 17 | **Conclusion** | In this study, we employed a bidirectional two‑sample MR framework to elucidate the genetic causal effects of PBC on IBD, and vice versa. The forward MR analysis provided compelling evidence of positive genetic effects of PBC on both UC and CD, establishing PBC as a shared genetic predisposing factor for both IBD subtypes. This finding carries clinical relevance: individuals diagnosed with PBC may warrant heightened vigilance and potentially routine screening for incident UC and CD. In the reverse direction, we observed a significant negative genetic effect of UC on PBC—suggesting a protective role—whereas no such effect was detected for CD on PBC. When IBD was considered as a composite phenotype, the positive effect of PBC on overall IBD remained consistent, while the effect of IBD on PBC was not supported. Collectively, these results delineate a directional and subtype‑specific genetic causality between PBC and IBD, with implications for risk stratification and clinical surveillance. |
|  | **Other information** |  |
| 18 | Conflict of Interest | The authors of this article declare that there are no conflicts of interest among them. |
| 19 | Funding | This work was financially supported by the Shaanxi Province Natural Science Foundation Research Program (No. 2025JC-YBQN-1266). |
| 20 | Data availability | This study employed openly accessible datasets sourced from the FinnGen consortium (https://www.finngen.fi/) and the IEU OpenGWAS database (https://gwas.mrcieu.ac.uk/). |

**Supplementary Table 2:** The data used in this study.

| **GWAS ID** | **Trait** | **Cases** | **Controls** | **SNPs** | **Population** |
| --- | --- | --- | --- | --- | --- |
| ebi-a-GCST005581 | Primary biliary cirrhosis | 2,861 | 8,514 | 119,756 | European |
| finn-b-K11_ULCER | Ulcerative colitis | 4,320 | 210,300 | 16,380,459 | European |
| finn-b-CHRONLARGE | Crohn's disease | 807 | 210,300 | 16,380,453 | European |
| finn-b-K11_IBD | Inflammatory bowel disease | 5673 | 213119 | 16380466 | European |

**Supplementary Table 3:** The instrumental variables used in MR analysis between exposure (primary biliary cirrhosis) and outcome (ulcerative colitis).

|  | **SNP** | **beta.exposure** | **se.exposure** | **pval.exposure** | **pval.outcome** | **palindromic** | **R2** | **F** |
| --- | --- | --- | --- | --- | --- | --- | --- | --- |
| 1 | rs10931468 | 0.319907 | 0.042373 | 4.36E-14 | 0.8703 | FALSE | 0.004986 | 56.98868 |
| 2 | rs11065979 | 0.183155 | 0.030841 | 2.87E-09 | 0.00985 | FALSE | 0.003091 | 35.2627 |
| 3 | rs11117431 | -0.23471 | 0.040941 | 9.88E-09 | 0.08603 | FALSE | 0.002881 | 32.8597 |
| 4 | rs12134279 | 0.248421 | 0.035805 | 3.97E-12 | 0.6026 | FALSE | 0.004214 | 48.1286 |
| 5 | rs12708716 | -0.23674 | 0.033056 | 7.97E-13 | 0.1093 | FALSE | 0.004489 | 51.28136 |
| 6 | rs1646019 | -0.27431 | 0.0354 | 9.28E-15 | 0.1628 | FALSE | 0.005251 | 60.03224 |
| 7 | rs17122453 | -0.32684 | 0.040557 | 7.71E-16 | 0.389 | FALSE | 0.005677 | 64.93086 |
| 8 | rs1800693 | 0.24059 | 0.03117 | 1.18E-14 | 0.2883 | FALSE | 0.00521 | 59.56616 |
| 9 | rs2267407 | 0.259283 | 0.035006 | 1.29E-13 | 0.001119 | FALSE | 0.0048 | 54.85253 |
| 10 | rs2293370 | -0.33729 | 0.041778 | 6.83E-16 | 0.944 | FALSE | 0.005698 | 65.16898 |
| 11 | rs2523882 | 0.227136 | 0.033713 | 1.61E-11 | 0.1606 | FALSE | 0.003975 | 45.38406 |
| 12 | rs3135024 | 0.506818 | 0.033803 | 8.10E-51 | 0.3897 | FALSE | 0.01938 | 224.7657 |
| 13 | rs34725611 | -0.20494 | 0.034886 | 4.24E-09 | 0.7895 | FALSE | 0.003025 | 34.50234 |
| 14 | rs35188261 | 0.416735 | 0.043315 | 6.52E-22 | 0.1086 | FALSE | 0.008072 | 92.54627 |
| 15 | rs522127 | -0.30897 | 0.031849 | 2.98E-22 | 0.004776 | FALSE | 0.008206 | 94.09466 |
| 16 | rs6871748 | -0.26371 | 0.035965 | 2.26E-13 | 0.087761 | FALSE | 0.004704 | 53.75269 |
| 17 | rs72678531 | 0.479954 | 0.037073 | 2.47E-38 | 0.03258 | FALSE | 0.014521 | 167.5761 |
| 18 | rs7665090 | 0.229162 | 0.030708 | 8.48E-14 | 0.05788 | FALSE | 0.004872 | 55.68229 |
| 19 | rs7775055 | 1.11317 | 0.074902 | 5.85E-50 | 0.02842 | FALSE | 0.019047 | 220.8285 |
| 20 | rs79513546 | -1.52005 | 0.212086 | 7.66E-13 | 0.4417 | FALSE | 0.004496 | 51.35886 |
| 21 | rs8067378 | 0.232058 | 0.030912 | 6.05E-14 | 8.03E-05 | FALSE | 0.00493 | 56.34447 |
| 22 | rs911263 | 0.227654 | 0.035199 | 9.95E-11 | 0.2912 | FALSE | 0.003664 | 41.82379 |

**Supplementary Table 4:** The instrumental variables used in MR analysis between exposure (primary biliary cirrhosis) and outcome (Crohn's disease).

|  | **SNP** | **beta.exposure** | **se.exposure** | **pval.exposure** | **pval.outcome** | **palindromic** | **R2** | **F** |
| --- | --- | --- | --- | --- | --- | --- | --- | --- |
| 1 | rs10931468 | 0.319907 | 0.042373 | 4.36E-14 | 0.002455 | FALSE | 0.004986 | 56.98868 |
| 2 | rs11065979 | 0.183155 | 0.030841 | 2.87E-09 | 0.2005 | FALSE | 0.003091 | 35.2627 |
| 3 | rs11117431 | -0.23471 | 0.040941 | 9.88E-09 | 0.2143 | FALSE | 0.002881 | 32.8597 |
| 4 | rs12134279 | 0.248421 | 0.035805 | 3.97E-12 | 0.002528 | FALSE | 0.004214 | 48.1286 |
| 5 | rs12708716 | -0.23674 | 0.033056 | 7.97E-13 | 0.6223 | FALSE | 0.004489 | 51.28136 |
| 6 | rs1646019 | -0.27431 | 0.0354 | 9.28E-15 | 0.2256 | FALSE | 0.005251 | 60.03224 |
| 7 | rs17122453 | -0.32684 | 0.040557 | 7.71E-16 | 0.1361 | FALSE | 0.005677 | 64.93086 |
| 8 | rs1800693 | 0.24059 | 0.03117 | 1.18E-14 | 0.8365 | FALSE | 0.00521 | 59.56616 |
| 9 | rs2267407 | 0.259283 | 0.035006 | 1.29E-13 | 0.01136 | FALSE | 0.0048 | 54.85253 |
| 10 | rs2293370 | -0.33729 | 0.041778 | 6.83E-16 | 0.3741 | FALSE | 0.005698 | 65.16898 |
| 11 | rs2523882 | 0.227136 | 0.033713 | 1.61E-11 | 0.2901 | FALSE | 0.003975 | 45.38406 |
| 12 | rs3135024 | 0.506818 | 0.033803 | 8.10E-51 | 0.5702 | FALSE | 0.01938 | 224.7657 |
| 13 | rs34725611 | -0.20494 | 0.034886 | 4.24E-09 | 0.7843 | FALSE | 0.003025 | 34.50234 |
| 14 | rs35188261 | 0.416735 | 0.043315 | 6.52E-22 | 0.095821 | FALSE | 0.008072 | 92.54627 |
| 15 | rs522127 | -0.30897 | 0.031849 | 2.98E-22 | 0.948 | FALSE | 0.008206 | 94.09466 |
| 16 | rs6871748 | -0.26371 | 0.035965 | 2.26E-13 | 0.629901 | FALSE | 0.004704 | 53.75269 |
| 17 | rs72678531 | 0.479954 | 0.037073 | 2.47E-38 | 0.3078 | FALSE | 0.014521 | 167.5761 |
| 18 | rs7665090 | 0.229162 | 0.030708 | 8.48E-14 | 0.9326 | FALSE | 0.004872 | 55.68229 |
| 19 | rs7775055 | 1.11317 | 0.074902 | 5.85E-50 | 0.055221 | FALSE | 0.019047 | 220.8285 |
| 20 | rs79513546 | -1.52005 | 0.212086 | 7.66E-13 | 0.2345 | FALSE | 0.004496 | 51.35886 |
| 21 | rs8067378 | 0.232058 | 0.030912 | 6.05E-14 | 0.3823 | FALSE | 0.00493 | 56.34447 |
| 22 | rs911263 | 0.227654 | 0.035199 | 9.95E-11 | 0.4067 | FALSE | 0.003664 | 41.82379 |
| Confounder | |  | | | | | | |
| 1 | rs11065979 | Lifetime smoking | | | | | | |

**Supplementary Table 5:** The instrumental variables used in MR analysis between exposure (ulcerative colitis) and outcome (primary biliary cirrhosis).

|  | **SNP** | **beta.exposure** | **se.exposure** | **pval.exposure** | **pval.outcome** | **palindromic** | **R2** | **F** |
| --- | --- | --- | --- | --- | --- | --- | --- | --- |
| 1 | rs1061537 | -0.1236 | 0.0238 | 2.13E-07 | 0.9532 | FALSE | 0.000126 | 26.96988 |
| 2 | rs10737481 | 0.1574 | 0.0225 | 2.76E-12 | 0.05598 | FALSE | 0.000228 | 48.93734 |
| 3 | rs10748781 | -0.1258 | 0.0237 | 1.18E-07 | 0.2711 | FALSE | 0.000131 | 28.17478 |
| 4 | rs11488194 | -0.259 | 0.0546 | 2.14E-06 | 0.03826 | FALSE | 0.000105 | 22.50143 |
| 5 | rs12088685 | -0.3034 | 0.0661 | 4.38E-06 | 0.1067 | FALSE | 9.82E-05 | 21.06804 |
| 6 | rs12946510 | 0.1272 | 0.0226 | 1.73E-08 | 8.02E-11 | FALSE | 0.000148 | 31.67767 |
| 7 | rs2836878 | -0.132 | 0.0262 | 4.79E-07 | 0.3714 | FALSE | 0.000118 | 25.3829 |
| 8 | rs3024493 | 0.1643 | 0.0312 | 1.34E-07 | 0.006303 | FALSE | 0.000129 | 27.73077 |
| 9 | rs3197999 | 0.1455 | 0.0231 | 3.14E-10 | 0.095801 | FALSE | 0.000185 | 39.67327 |
| 10 | rs36051895 | 0.1203 | 0.0238 | 4.29E-07 | 0.7052 | FALSE | 0.000119 | 25.54896 |
| 11 | rs4676410 | 0.1561 | 0.0255 | 9.07E-10 | 0.4887 | FALSE | 0.000175 | 37.47325 |
| 12 | rs6671847 | -0.1254 | 0.0227 | 3.26E-08 | 0.7047 | FALSE | 0.000142 | 30.51682 |
| 13 | rs7936070 | 0.1128 | 0.0228 | 7.64E-07 | 0.1084 | FALSE | 0.000114 | 24.47623 |

**Supplementary Table 6:** The instrumental variables used in MR analysis between exposure (Crohn's disease) and outcome (primary biliary cirrhosis).

|  | **SNP** | **beta.exposure** | **se.exposure** | **pval.exposure** | **pval.outcome** | **palindromic** | **R2** | **F** |
| --- | --- | --- | --- | --- | --- | --- | --- | --- |
| 1 | rs10748781 | -0.2486 | 0.0535 | 3.37E-06 | 0.2711 | FALSE | 0.000102 | 21.59189 |
| 2 | rs12119179 | 0.2596 | 0.0522 | 6.62E-07 | 0.4893 | FALSE | 0.000117 | 24.73229 |
| 3 | rs12374574 | 0.3014 | 0.0648 | 3.34E-06 | 0.9934 | FALSE | 0.000102 | 21.63378 |
| 4 | rs7107372 | 0.3041 | 0.0621 | 9.70E-07 | 0.9683 | FALSE | 0.000114 | 23.9798 |

**Supplementary Table 7: MR analysis process of exposure and outcome.**

| **Exposure** | **Outcome** | **Heterogeneity Test** | | **Pleiotropy Test** | **MR-PRESSO** | | **MR-RAPS** |
| --- | --- | --- | --- | --- | --- | --- | --- |
|  |  | **Cochran’s Q Test (IVW)** | **Rucker’s**  **Q Test (MR-Egger)** | **Egger**  **Intercept**  **(MR-Egger)** | **Distortion Test**  **(Outliers)** | **Global**  **Test**  **(Pleiotropy)** | **Normal**  **Distribution** |
|  |  | **P value** | **P value** | **P value** | **Number** | **P value** | **P value** |
| PBC | UC 1 | 1.73E-05 | 6.59E-05 | 0.167 | 2 | <0.001 | 0.882 |
| PBC | UC 2 | 0.015 | 0.036 | 0.114 | 1 | 0.026 | 0.894 |
| PBC | CD 1 | 0.021 | 0.015 | 0.795 | 1 | 0.029 | 0.697 |
| UC | PBC 1 | 0.008 | 0.023 | 0.167 | 1 | 0.013 | 0.587 |
| UC | PBC 2 | 0.107 | 0.388 | 0.039 | 0 | 0.107 | 0.496 |

MR: mendelian randomization; PBC: Primary biliary cirrhosis; UC: Ulcerative colitis; CD: Crohn's disease.

**Supplementary Table 8:** The instrumental variables used in MR analysis between exposure (primary biliary cirrhosis) and outcome (inflammatory bowel disease).

|  | **SNP** | **beta.exposure** | **se.exposure** | **pval.exposure** | **pval.outcome** | **palindromic** | **R2** | **F** |
| --- | --- | --- | --- | --- | --- | --- | --- | --- |
| 1 | rs10931468 | 0.319907 | 0.0423732 | 4.36114e-14 | 0.8992 | FALSE | 0.004985891 | 56.98867954 |
| 2 | rs11117431 | -0.23471 | 0.0409413 | 9.87598e-09 | 0.0627899 | FALSE | 0.002880949 | 32.85969768 |
| 3 | rs12134279 | 0.248421 | 0.0358054 | 3.97375e-12 | 0.1871 | FALSE | 0.004213996 | 48.12859521 |
| 4 | rs12708716 | -0.236736 | 0.0330557 | 7.9671e-13 | 0.0350598 | FALSE | 0.004488804 | 51.28135921 |
| 5 | rs1646019 | -0.275753 | 0.0353998 | 6.71738e-15 | 0.2177 | FALSE | 0.005306118 | 60.66839546 |
| 6 | rs17122453 | -0.326839 | 0.0405574 | 7.71259e-16 | 0.0624195 | FALSE | 0.005676801 | 64.93086191 |
| 7 | rs1800693 | 0.24059 | 0.0311702 | 1.17598e-14 | 0.4281 | FALSE | 0.005210218 | 59.56615954 |
| 8 | rs2293370 | -0.337293 | 0.0417781 | 6.83439e-16 | 0.8604 | FALSE | 0.005697501 | 65.16897945 |
| 9 | rs2523882 | 0.227136 | 0.0337129 | 1.61287e-11 | 0.3567 | FALSE | 0.003974648 | 45.38406116 |
| 10 | rs3135024 | 0.506818 | 0.0338025 | 8.09841e-51 | 0.7795 | FALSE | 0.019380087 | 224.7657079 |
| 11 | rs34725611 | -0.204935 | 0.0348862 | 4.24405e-09 | 0.7085 | FALSE | 0.003024531 | 34.50234182 |
| 12 | rs35188261 | 0.416735 | 0.0433154 | 6.52379e-22 | 0.0439097 | FALSE | 0.008071684 | 92.54627195 |
| 13 | rs6871748 | -0.263705 | 0.035965 | 2.26308e-13 | 0.107 | FALSE | 0.004704109 | 53.75269375 |
| 14 | rs7665090 | 0.229162 | 0.0307076 | 8.47618e-14 | 0.1605 | FALSE | 0.004872153 | 55.68229053 |
| 15 | rs7775055 | 1.11317 | 0.0749024 | 5.8479e-50 | 0.00120601 | FALSE | 0.019047075 | 220.828516 |
| 16 | rs79513546 | -1.52005 | 0.212086 | 7.65597e-13 | 0.4776 | FALSE | 0.004495557 | 51.35886041 |
| 17 | rs911263 | 0.227654 | 0.0351986 | 9.95176e-11 | 0.7791 | FALSE | 0.003663989 | 41.82379379 |

**Supplementary Table 9:** The instrumental variables used in MR analysis between exposure (inflammatory bowel disease) and outcome (primary biliary cirrhosis).

|  | **SNP** | **beta.exposure** | **se.exposure** | **pval.exposure** | **pval.outcome** | **palindromic** | **R2** | **F** |
| --- | --- | --- | --- | --- | --- | --- | --- | --- |
| 1 | rs10737481 | 0.1229 | 0.0199 | 6.20197e-10 | 0.0559796 | FALSE | 0.000174297 | 38.14113767 |
| 2 | rs1156534 | -0.1418 | 0.026 | 4.95998e-08 | 0.6405 | FALSE | 0.00013593 | 29.74416597 |
| 3 | rs17689550 | -0.134 | 0.0245 | 4.68198e-08 | 0.1933 | FALSE | 0.000136706 | 29.91392897 |
| 4 | rs2230724 | 0.1094 | 0.0199 | 4.12297e-08 | 0.4475 | FALSE | 0.000138114 | 30.22209186 |
| 5 | rs3197999 | 0.131 | 0.0204 | 1.38099e-10 | 0.095801 | FALSE | 0.000188438 | 41.23616669 |
| 6 | rs4409764 | -0.1183 | 0.0199 | 2.59102e-09 | 0.3213 | FALSE | 0.000161496 | 35.33941585 |
| 7 | rs4676410 | 0.1414 | 0.0225 | 3.15798e-10 | 0.4887 | FALSE | 0.000180478 | 39.49388095 |
| 8 | rs7936070 | 0.116 | 0.0201 | 8.11503e-09 | 0.2385 | FALSE | 0.000152204 | 33.30580183 |
| 9 | rs9988642 | -0.2865 | 0.0487 | 4.10904e-09 | 0.0382596 | FALSE | 0.000158158 | 34.60886527 |

**Supplementary Figure 1:** Forward MR analysis process, primary biliary cirrhosis as the exposure variable, ulcerative colitis and Crohn's disease as the outcome of interest. The investigation utilized a comprehensive array of methodologies, comprising random-effects IVW, MR Egger, weighted median, simple mode, weighted mode, maximum likelihood, penalized weighted median, and fixed-effects IVW.

**Supplementary Figure 2:** The first round of forward MR analysis, the primary biliary cirrhosis as exposure and the ulcerative colitis as the outcome. A. scatter plot; B. MR radial plots; C. leave-one-out analysis; D. normal distribution.

**Supplementary Figure 3:** The second round of forward MR analysis, the primary biliary cirrhosis as exposure and the ulcerative colitis as the outcome. A. scatter plot; B. MR radial plots; C. leave-one-out analysis; D. normal distribution.

**Supplementary Figure 4:** The first round of forward MR analysis, the primary biliary cirrhosis as exposure and the Crohn's disease as the outcome. A. scatter plot; B. MR radial plots; C. leave-one-out analysis; D. normal distribution.

**Supplementary Figure 5:** Reverse MR analysis process, ulcerative colitis as the exposure variable, primary biliary cirrhosis as the outcome of interest. The investigation utilized a comprehensive array of methodologies, comprising random-effects IVW, MR Egger, weighted median, simple mode, weighted mode, maximum likelihood, penalized weighted median, and fixed-effects IVW.

**Supplementary Figure 6:** The first round of reverse MR analysis, the ulcerative colitis as exposure and the primary biliary cirrhosis as the outcome. A. scatter plot; B. MR radial plots; C. leave-one-out analysis; D. normal distribution.

**Supplementary Figure 7:** The second round of reverse MR analysis, the ulcerative colitis as exposure and the primary biliary cirrhosis as the outcome. A. scatter plot; B. MR radial plots; C. leave-one-out analysis; D. normal distribution.
